# Prices and Affordability of Essential Medicines in 72 Low-, Middle-, and High-Income Markets

**DOI:** 10.1001/jamahealthforum.2025.2043

**Published:** 2025-08-15

**Authors:** Olivier J. Wouters, Cyprien Denolle, Jinru Wei, Irene Papanicolas

**Affiliations:** 1 Department of Health Services, Policy and Practice, Brown University School of Public Health, Providence, Rhode Island; 2 Department of Health Policy, London School of Economics and Political Science, London, United Kingdom

## Abstract

**Importance:**

Little is known about how the prices and affordability of medicines included on the World Health Organization’s Model List of Essential Medicines vary across the globe.

**Objective:**

To compare the list prices and affordability of essential medicines across high-, middle-, and low-income markets.

**Design and Setting:**

This cross-sectional study examined data from 2022 on list prices and volumes of 549 essential medicines in 72 high-, middle-, and low-income markets (covering 87 countries). These data were obtained from IQVIA. The statistical analyses were performed between August 2024 and March 2025.

**Main Outcomes and Measures:**

Laspeyres price indices were used to compare average drug prices across countries, both in nominal and purchasing power parity–adjusted terms. The affordability of 8 essential medicines, used to treat major causes of death and disability globally, was assessed by calculating how many days of minimum wage would be required to pay for 1 month of treatment.

**Results:**

The availability of essential medicines ranged from 225 (41%) in Kuwait to 438 (80%) in Germany (base country). After accounting for purchasing power parities, prices of essential medicines in Lebanon were, on average, 18.1% of those in Germany (Lebanon price index, 18.1 vs Germany price index, 100), while average prices in the US were 3.0 times higher than in Germany (US price index, 298.2). A positive association was observed between countries’ gross domestic product per capita (expressed in logarithmic terms) and nominal drug prices (*R* = 0.30; *P* = .01), indicating that richer countries generally had higher drug prices. However, when adjusting for the purchasing power of different currencies, an inverse association was observed (*R* = −0.35; *P* = .003), suggesting that richer countries had lower real prices. Drug affordability, as measured by the number of days’ minimum wage needed to purchase a month’s treatment, varied widely, with median affordability highest in Europe and the Western Pacific, and lowest in Africa and Southeast Asia.

**Conclusions and Relevance:**

The results of this cross-sectional analysis showed significant variation in the prices and affordability of 549 essential medicines across 72 markets in 2022. Strategies to promote equitable drug prices and improve drug affordability are urgently needed.

## Key Points

**Question:**

How do the list prices and affordability of essential medicines compare across high-, middle-, and low-income countries?

**Findings:**

This cross-sectional study, which analyzed data from 2022 on list prices and volumes of 549 essential medicines in 72 high-, middle-, and low-income markets, found large differences in list prices and affordability of medicines across markets. When adjusting for the purchasing power of different currencies, there was an inverse association between drug prices and country income levels, suggesting that richer countries had lower real prices.

**Meaning:**

Strategies to promote equitable drug prices and improve drug affordability are urgently needed.

## Teaser

This cross-sectional study compares the list prices and affordability of essential medicines across high-, middle-, and low-income markets.

## Introduction

Equitable access to essential medicines, which the World Health Organization (WHO) defines as those that meet the priority needs of the population,^1^ is key to achieving universal health care coverage.^2^ However, it is estimated that essential medicines are unaffordable or unavailable to 1 in 4 people worldwide.^3^ As governments strive to achieve universal access to essential medicines, it is important to understand how the prices and affordability of these medicines vary between countries. High drug prices strain both personal and government budgets, especially in low- and middle-income countries, where patients often face high out-of-pocket costs.

Although many national surveys of medicine prices and affordability have been conducted, fewer analyses have compared these variables internationally, with most research dating back to 2011 or earlier.^4,5,6,7,8,9,10,11,12,13,14,15,16,17,18,19,20^ Many of these studies have focused on high-income countries. Of the studies that also included low- and middle-income nations, most compared the prices of 15 or fewer medicines or included a small number of countries, limiting the generalizability of those findings. Therefore, little is known about the relationship between national income and drug prices. The question remains whether some poor countries routinely pay more than rich countries for the same prescription drugs, possibly owing to weaker pharmaceutical pricing policies.

This cross-sectional study used global data on pharmaceutical sales to compare the list prices of 549 essential medicines in 72 high-, middle-, and low-income markets (covering 87 countries) in 2022, both in nominal and purchasing power–adjusted terms. We also evaluated the affordability of 8 essential medicines used to treat major causes of death and disability globally.

## Methods

Institutional review board approval was not required for this study because no data were collected from human participants. We followed the Strengthening the Reporting of Observational Studies in Epidemiology (STROBE) reporting guideline.

### Sample Identification

We used the 22nd edition of the WHO Model List of Essential Medicines^1^ to define our basket of essential medicines. This list included both newer, patent-protected medicines (ie, single-source brand-name drugs) and older medicines available in generic form (ie, multisource drugs). If the WHO identified alternative treatments to a medicine in the Essential Medicines List, we included both the primary therapy and its alternatives. For example, the WHO lists mesalazine as an alternative to the anti-inflammatory drug sulfasalazine. Similarly, all angiotensin-converting enzyme inhibitors are recognized as alternatives to enalapril.

We excluded products without an ATC (anatomical therapeutic chemical) code, as well as products belonging to the following categories: antidotes, antivenoms and antitoxins, antiseptics and disinfectants, blood products of human origin and plasma substitutes, dental preparations, diagnostic agents, dialysis solutions, medical gases (ie, oxygen), solutions correcting water, electrolyte, and acid-base disturbances, vaccines, and vitamins and minerals. Products in these categories are generally priced and procured differently from other products on the Essential Medicines List.

We also excluded antihelminthics, antituberculosis drugs, antileprosy drugs, antiprotozoal drugs (including antimalarials), and HIV/AIDS drugs, as these products are often procured by international agencies outside the normal commercial channels tracked by IQVIA.

These restrictions left us with a sample of 549 essential medicines. For each of these products, we obtained 2022 data from IQVIA on sales in 72 markets (covering 87 countries): 69 individual countries, the Hong Kong special administrative region, and 2 regional groups of countries (Central America and West Africa). IQVIA aggregates national data for 6 countries in Central America (Costa Rica, El Salvador, Guatemala, Honduras, Nicaragua, and Panama) and 12 in West Africa (Benin, Burkina Faso, Cameroon, Chad, Republic of Congo, Côte d’Ivoire, Gabon, Guinea, Mali, Niger, Senegal, and Togo). eAppendix 1 in Supplement 1 lists all medicines and markets in our sample.

Sales were recorded in terms of volume and monetary value (at the retail pharmacy level, which includes any distribution or pharmacy fees). The data did not capture confidential rebates and discounts. Volumes were measured in the number of counting units, which IQVIA defined as the smallest unit in which a medicine could be produced (eg, 1 tablet or 1 mL of liquid). The dataset captured all strengths and formulations of different active ingredients, which in some cases included formulation-strength combinations not listed on the WHO Model List of Essential Medicines.

All monetary values were expressed in local currency units. IQVIA converted these amounts to US dollars using spot exchange rates. Additionally, we converted each local currency amount to international dollars using World Bank purchasing power parity conversion factors, based on gross domestic product (GDP). For Central America and West Africa, we selected 1 country from each region, Costa Rica and Côte d’Ivoire, because they were the most populous countries with available minimum wage data at the time of initial analysis, which were needed for the affordability calculations. The US dollar amounts for the 2 regions were first converted into local currencies (West African CFA francs for Côte d’Ivoire and colones for Costa Rica). We then converted these amounts into international dollars based on the conversion factors for these 2 countries.

### Medicine Prices

To compare average drug prices internationally, we calculated Laspeyres price indices, which attach greater weight to the prices of drugs that were more widely consumed, as done in earlier studies.^8,9,16,21,22,23^

First, for each essential medicine, we calculated the average list price per unit in each country. This was done by dividing total retail sales by the number of units sold. Second, we calculated indices based on utilization patterns in a base country. We selected Germany as the base country, as it was the country with the most essential medicines available. By weighting prices by volumes, the prices of highly consumed essential medicines were given greater consideration. The index for a given country was calculated as:
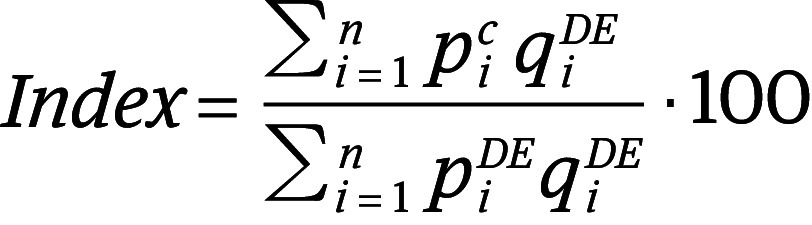
where *p* is the list unit price of an essential medicine (*i*) in a comparator country (*c*) or Germany (*DE*), and *q* is the corresponding quantity in doses. We constructed 2 versions of the Laspeyres indices, one using purchasing power parity–adjusted retail sales, which we treated as the base-case results, and another using retail sales in US dollars based on nominal exchange rates.

The Laspeyres index calculation may assign a large weight to a medicine that is heavily consumed in the base country (Germany) but that is not consumed much in a comparator country. To minimize such biases, for each bilateral comparison, we excluded the top 1% of medicines with the largest differences in the ratio of volume in Germany to volume in the comparator country.

We also broke down the results by clinical category. We produced indices for 7 categories of essential medicines used to treat the leading causes of death and disability globally, according to the 2019 Global Burden of Disease study: cancer, cardiovascular disease, diabetes, hepatitis B and C, asthma and chronic obstructive pulmonary disease, mental health disorders, and pain (including migraine).^24^ We included antibiotics as a separate category in our analysis, given their clinical importance. The products included in each of the categories are shown in eAppendices 1 and 2 in Supplement 1.

Spearman rank correlations were used to examine the association between Laspeyres indices and GDP per capita (measured on a logarithmic scale) in 2022, or the nearest available year. Data on GDP per capita were sourced from the World Bank. Data from Costa Rica were used as a proxy for the Central America country group, and data from Côte d’Ivoire for the West Africa country group.

### Medicine Utilization

We calculated the proportion of essential medicines that were sold in generic vs brand-name form in each country (based on volume and monetary value). We distinguished between brand-name, generic, and over-the-counter drugs.

### Medicine Affordability

To measure the affordability of medicines in each country, we estimated the number of days’ minimum wage needed to pay for 1 month of treatment based on nominal prices, based on a method developed by the WHO and Health Action International.^25^ The WHO/Health Action International method uses daily wage of the lowest-paid unskilled government worker to measure affordability, whereas we relied on minimum wage data, which were more widely available. Data on national minimum wages were sourced from the International Labour Organization (eAppendix 3 in Supplement 1). As done for the pricing calculations, wages for Côte d’Ivoire and Costa Rica were used as proxies for West Africa and Central America, respectively.

We estimated the affordability of key medicines for the 7 major causes of death and disability mentioned previously. For each condition, we selected the drug available in the greatest number of countries; if 2 or more drugs were available in the same number of countries, we picked the medicine with the highest global consumption (volume). We also included the most widely used antibiotic on the WHO’s list. These were amoxicillin (antibiotic used to treat community-acquired pneumonia), escitalopram (depression), ibuprofen (pain), short-acting insulin as a soluble insulin injection (diabetes), losartan (ischemic heart disease), paclitaxel (lung cancer; the product is also used to treat other cancers), salbutamol (asthma and chronic obstructive pulmonary disease), and tenofovir disoproxil (hepatitis B).

We used international clinical guidelines to outline standard treatment regimens for each drug (eAppendix 3 in Supplement 1). We verified the treatment regimens with board-certified clinicians and pharmacists.

### Statistical Analysis

We used 2-sided statistical tests, with *P* < .05 considered significant. The data were analyzed using Python, version 3.11.4 (Python Software Foundation), and R statistical software, version 4.4.0 (R Project for Statistical Computing). The statistical analyses were performed between August 2024 and March 2025.

## Results

### Descriptive Statistics

The dataset included the 2022 list prices and volumes of 549 essential medicines in 72 markets. These comprised 40 high-income markets (covering 39 countries plus Hong Kong), 32 middle-income markets (covering 42 countries), and 1 low-income market (covering 6 countries). The West Africa market included both low- and middle-income countries and was counted in both categories; therefore, the income group breakdown totals 73 entries. The data captured countries in all WHO regions, ranging from 13 of 47 countries in the WHO African region (28%) to 33 of 53 countries in the European region (62%) (Table 1). Availability of these 549 medicines, measured in terms of whether any sales were recorded in 2022, varied considerably across markets and regions. Germany had the highest availability (438 of 549 [80%]), and Kuwait the lowest (225 of 549 [41%]) (eAppendix 4 in Supplement 1). The Western Pacific region had the highest median number of drugs available (381 of 549 [69%]), and the Eastern Mediterranean region the lowest (323 of 549 [59%]) (Table 1).

**Table 1.** Essential Medicines Spending and Utilization by WHO Region, 2022^a^

Variable	WHO region					
	African	Americas	Eastern Mediterranean	Europe	Southeast Asia	Western Pacific
No. of countries included (% of region)	13 (28)^b^	17 (49)^c^	9 (43)	33 (62)	5 (45)	10 (37)^d^
No. of essential medicines available of 549 in the sample						
Median	359	347	323	367	345	381
IQR	350-367	319-382	287-330	342-390	308-377	358-394
Annual spending on essential medicines, millions of $						
Median	1463	1008	1067	2028	1825	1886
IQR	1289-1637	687-7051	271-1698	687-4771	1600-3134	1144-6102
Annual spending on essential medicines per capita, $						
Median	35	54	39	192	7	122
IQR	33-37	39-132	28-49	124-303	7-9	34-156
No. of doses consumed per capita						
Median	402	245	275	634	143	371
IQR	384-420	139-419	175-386	554-753	114-179	227-641
Generic market share by volume, %						
Median	80	69	71	63	82	60
IQR	77-83	64-77	69-75	54-70	78-90	48-69
Generic market share by value, %						
Median	67	63	60	32	80	28
IQR	62-72	57-71	51-63	29-37	70-84	24-48
GDP per capita, $						
Median	1515	10 111	4295	27 227	3343	32 160
IQR	831-2492	6477-15 411	3608-30 448	18 356-49 942	2688-4788	12 328-48 522
Annual health expenditure per capita, $						
Median	57	611	299	2499	161	3260
IQR	40-81	417-1415	221-1442	1159-5738	74-166	487-4347
Health expenditure as share of GDP, %						
Median	4.35	9.34	5.78	9.38	3.71	5.87
IQR	3.76-5.56	7.57-9.71	5.31-6.97	7.39-11.04	3.28-4.07	5.38-10.05

Abbreviations: GDP, gross domestic product; WHO, World Health Organization.

^a^ All data were sourced from IQVIA (based on retail sales), except GDP per capita and health expenditure, sourced from the World Bank. All data are from 2022, except health expenditure (2021) and health expenditure per capita (2021).

^b^ Data for 12 countries were aggregated as West Africa.

^c^ Data for 6 countries were aggregated as Central America.

^d^ The analysis included 10 of 27 countries (37%) in the Western Pacific region, and the Hong Kong market was considered part of this region, amounting to 11 markets total.

Annual spending on essential medicines varied across regions. The European region had the highest median (IQR) spending in total ($2.0 billion [$0.7 billion-$4.8 billion]) and per capita ($192 [$124-$303]). The Americas region had the lowest median (IQR) total spend ($1 billion [$0.7 billion-$7.1 billion]), while the Southeast Asia region had the lowest median (IQR) spending per capita ($7 [$7-$9]).

The median (IQR) number of doses of essential medicines consumed per capita in 2022 was highest in Europe (634 [554-753]) and lowest in Southeast Asia (143 [114-179]). Generics (including biosimilars, where relevant) accounted for the majority of market share in terms of the number of doses consumed. The median (IQR) generic market share (by volume) ranged from 60% (48%-69%) in the Western Pacific region to 82% (78%-90%) in the Southeast Asia region. Generics accounted for a smaller market share in monetary terms (by value). Additional country statistics can be found in eAppendix 4 in Supplement 1.

### Medicine Prices

**Figure 1.**
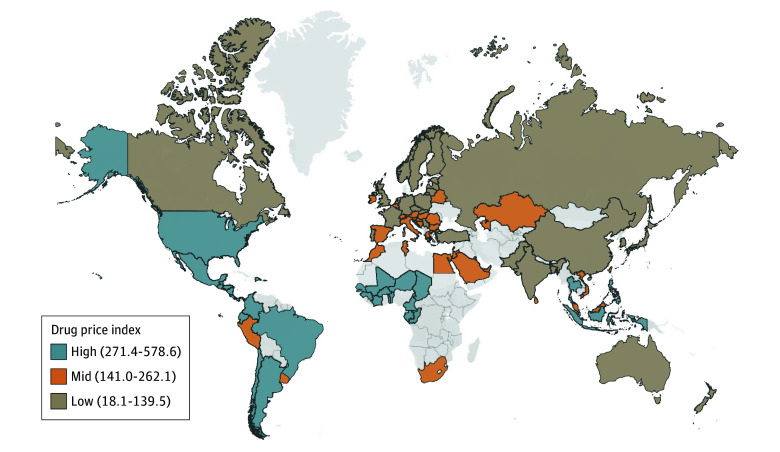
Average Prices of Essential Medicines in 87 Low-, Middle-, and High-Income Countries in 2022, With Countries Grouped by Tercile The indices were calculated based on purchasing power parity–adjusted prices. Data were available for 69 individual countries, plus Hong Kong. Data for an additional 6 countries were aggregated as Central America, and data for 12 countries were aggregated as West Africa. Thus, the sample consisted of 72 markets, covering 87 countries (69 individual countries and 2 regions of 18 countries) plus the special administrative region of Hong Kong.

After adjusting for differences in purchasing power parities, average drug prices varied widely across markets (Figure 1). Laspeyres price indices ranged from 18.1 in Lebanon to 578.6 in Argentina, meaning prices in Lebanon were on average 18.1% of those in the base country (Germany price index, 100), while average prices were approximately 5.8 times higher in Argentina than in Germany. In contrast, average prices in the US were 3.0 times higher than in Germany (US price index, 298.2). Across regions, median (IQR) Laspeyres price indices were, from least to greatest, as follows: 132.1 (96.4-176.2) for the Western Pacific region, 138.7 (121.1-162.2) for the European region, 165.3 (139.2-393.2) for the Southeast Asia region, 201.1 (188.3-224.9) for the Eastern Mediterranean region, 227.9 (not evaluable) for the African region (n = 2 markets), and 311.4 (255.6-392.1) for the Americas region (eAppendix 5 in Supplement 1).

Based on purchasing power parity–adjusted prices, 2 of 2 markets in the African region, 12 of 12 in the Americas region, 7 of 9 in the Eastern Mediterranean region (78%), 28 of 33 in the European region (85%), 4 of 5 in the Southeast Asia region (80%), 8 of 11 in the Western Pacific region (73%) had Laspeyres indices above 100, indicating prices for these countries were, on average, higher than in Germany.

Drug prices also varied considerably by disease area between markets (eAppendix 5 in Supplement 1). For most countries, the highest-priced drugs (relative to Germany) tended to be those to treat patients with mental and behavioral disorders (median index ≥110 in all regions) and cardiovascular disease medications (median index ≥130 in all regions), while the lowest-priced products were those to treat hepatitis B and C (median index ≤100 in all regions) (eAppendix 5 in Supplement 1).

**Figure 2.**
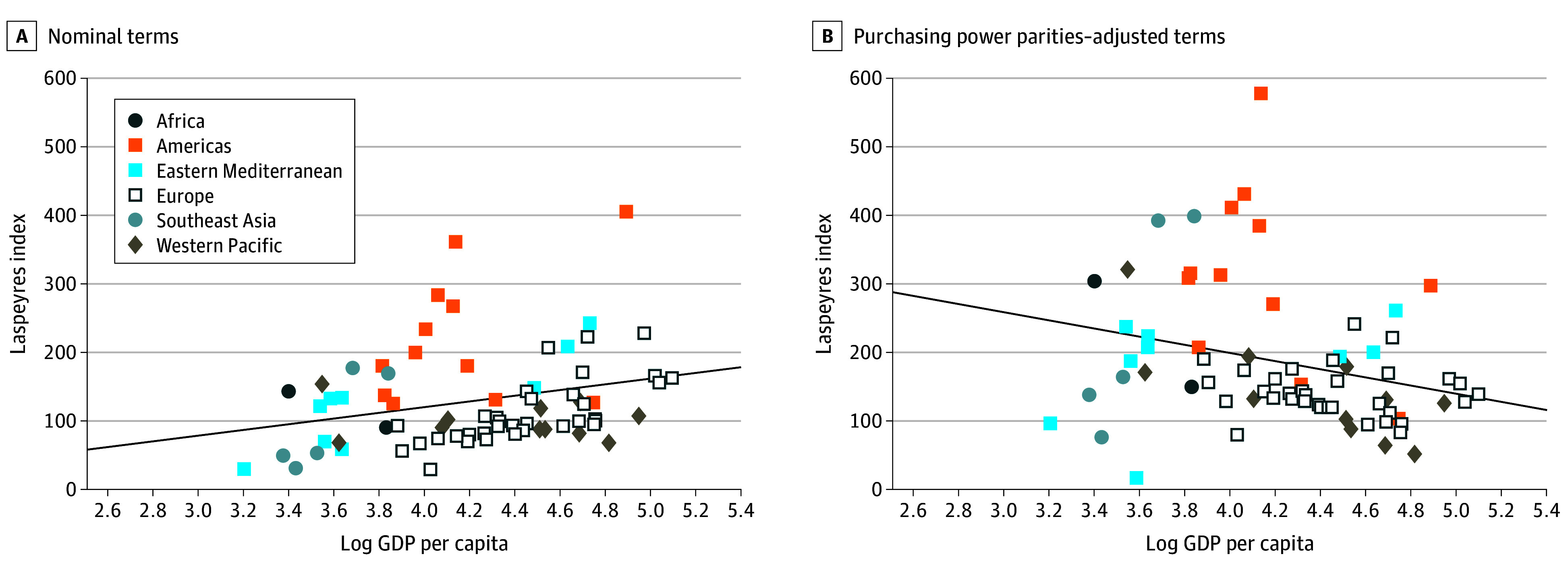
Association of Gross Domestic Product (GDP) Per Capita With Average Drug Prices, 2022

Results varied depending on whether nominal or purchasing power parity–adjusted prices were used (eAppendices 5 and 6 in Supplement 1). For example, the US had the highest price index in in nominal terms (407.5), but not after adjusting for differences in purchasing power parities (298.2). Although Pakistan had the lowest prices in nominal terms (30.3), prices were closer to those in Germany when purchasing power parities were accounted for (97.6). The comparison of price indices at the country level with the logarithm of national GDP per capita showed a significant positive correlation when nominal exchange rates were used to convert prices (*R* = 0.30; *P* = .01) (Figure 2), suggesting that richer countries faced higher nominal prices for essential medicines. When applying purchasing power parities to convert the prices, the association between price indices and the logarithm of GDP per capita was reversed, showing a significant negative correlation between the two (*R* = −0.35; *P* = .003). This suggests that richer countries faced lower real prices when accounting for differences in purchasing power.

### Medicine Utilization

**Figure 3.**
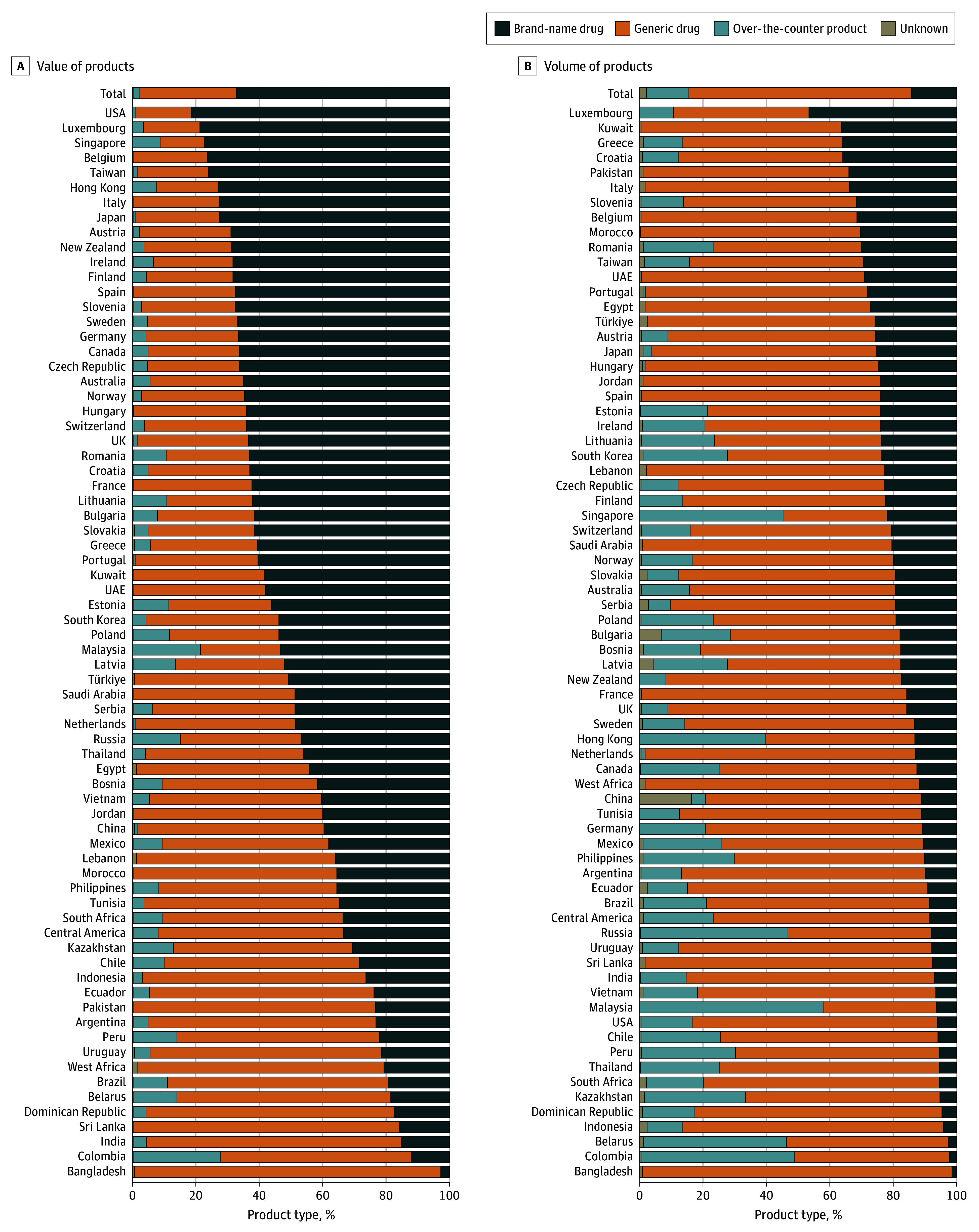
Breakdown of Brand-Name, Generic, and Over-the-Counter Drugs in Each Market by Value and Volume, 2022 Data for 12 countries were aggregated as West Africa, and data for 6 countries were aggregated as Central America. UAE indicates United Arab Emirates.

The composition of the basket of essential medicines, in terms of brand-name, generic, and over-the-counter products, also varied by market (Figure 3; eAppendix 5 in Supplement 1). Brand-name products made up $420 billion of $624 billion (67%) of the basket value across all countries, while generic drugs made up $189 billion (30%), and over-the-counter products $14 billion (2%), with $1 billion unknown (<1%). Brand-name products made up 232 billion of 1617 billion counting units, a 14% share of total volume of medicines across all countries, while generics made up 1133 billion counting units (70%), and over-the-counter products 218 billion (13%), with 34 billion unknown (2%). (Percentages do not add up to 100% due to rounding.)

There was variation in the composition of the volume and value of the different types of products across markets. Figure 3 shows markets ranked by brand-name drug market shares.

### Medicine Affordability

Data on minimum wages were available for 65 of the 72 markets in the sample. Levels of affordability varied considerably across country income levels (Table 2**)**. The most affordable essential medications were amoxicillin, ibuprofen, and salbutamol, all 3 of which cost less than 1.2 days’ minimum wage across all study countries (eAppendix 5 in Supplement 1); amoxicillin and ibuprofen both had shorter treatment durations. The least affordable treatment was paclitaxel; for example, the median (IQR) number of days’ minimum wage needed to pay for paclitaxel in lower-middle–income countries was 40.9 (24.1-73.1) days (Table 2). Median affordability was highest in Europe and the Western Pacific, and lowest in Africa and Southeast Asia (eAppendix 5 in Supplement 1).

**Table 2.** Days of Minimum Wage Required to Pay for a Course of Treatment by Country Income Level, 2022^a^

Income level		No. of days of minimum wage required to pay for a course of treatment							
		Amoxicillin	Escitalopram	Ibuprofen	Insulin injection (soluble)	Losartan	Paclitaxel	Salbutamol	Tenofovir disoproxil
Lower-middle–income countries	Median	0.42	1.48	0.19	0.39	0.81	40.93	0.18	4.06
	IQR	0.28-0.55	1.20-2.37	0.11-0.25	0.22-0.76	0.64-1.33	24.09-73.05	0.10-0.29	3.19-5.67
	Range	0.05-0.87	0.30-6.18	0.04-0.38	0.03-2.53	0.32-3.09	8.02-129.54	0.01-0.49	0.95-10.04
Upper-middle–income countries	Median	0.27	2.18	0.16	0.43	0.38	12.96	0.12	1.95
	IQR	0.15-0.53	1.39-2.94	0.11-0.43	0.36-0.54	0.27-0.52	4.81-16.97	0.09-0.17	0.96-4.87
	Range	0.10-1.00	0.14-6.05	0.06-1.17	0.11-1.48	0.12-2.37	1.98-302.13	0.04-0.48	0.57-12.14
High-income countries	Median	0.09	0.23	0.08	0.17	0.12	4.86	0.03	3.54
	IQR	0.05-0.13	0.11-0.28	0.05-0.15	0.11-0.23	0.09-0.14	1.99-9.24	0.02-0.04	1.72-6.03
	Range	0.02-0.80	0.01-3.46	0.01-0.35	0.06-0.77	0.01-2.58	0.38-36.85	0.01-0.19	0.31-13.22

^a^ Wages for Côte d’Ivoire and Costa Rica were used as proxies for West Africa and Central America, respectively. Therefore, no results were reported for low-income markets.

## Discussion

The results of this cross-sectional study showed wide variation in the prices of 549 essential medicines in 2022 across 72 markets. List prices of essential medicines were highest, on average, in the Americas and lowest in the Western Pacific after accounting for differences in purchasing power parities. The availability of essential medicines varied across regions, with markets in the Eastern Mediterranean region having the lowest median availability. When examining the relationship between prices and GDP per capita, we found that nominal prices were higher in richer countries. However, after adjusting for purchasing power, we found that real prices were higher in poorer countries. There was also substantial variation in the number of days’ minimum wage needed to purchase 8 essential medicines used to treat prevalent conditions worldwide, with treatments generally less affordable in poorer countries.

Our results offer insights for national policymakers and international health care organizations aiming to expand medicine coverage by mapping inequality in drug availability, prices, and affordability globally. Our results show that while drugs may have a lower nominal price in low- and middle-income countries, they may still be less affordable when considering the relative purchasing power of local currencies. This indicates that some poorer countries face a higher burden of medication costs, even if the price for the same medicine is lower compared to richer countries. For example, while prices in India were low in nominal terms (fourth lowest of 72 markets), they were toward the middle of the range when expressed in purchasing power parity–adjusted terms (29th of 72 markets). What’s more, the cost burden faced by governments and patients in low- and middle-income countries is probably more inequitable than our findings suggest. In high-income countries, a large share of drug costs is subsidized by the state, which reduces the direct financial burden on patients for prescription medicines.^26^ By contrast, the out-of-pocket burden in low- and middle-income countries is generally much larger.

Our findings are consistent with those of 2 previous studies,^13,27^ both of which reported that certain low- and middle-income countries paid higher prices for a number of medicines than high-income countries. Further work is needed to explore differences in the pricing and affordability of treatments within countries. One analysis found large variation in the availability and affordability of blood-pressure–lowering medicines across rural and urban areas in some countries.^10^ It is also important to study the impact of retail markups and taxation on medicines, which can be important in some settings.^28^

Several factors may explain the cross-country disparities highlighted by this analysis. Rich countries may have more effective policies in place to control the prices and utilization of medicines than poor countries. This includes health technology assessment for brand-name drugs and internal reference pricing for generic drugs, alongside demand-side measures, like generic prescribing and substitution policies. High-income countries may also have greater leverage in negotiations with drug companies over the prices of novel therapies as they represent more commercially attractive markets.

The high price tags of many medicines, coupled with inequitable access to medicines both within and across countries, have fueled a global debate about fair drug prices.^29,30,31^ Governments have a range of options to rein in drug prices. One strategy is for countries to join forces to negotiate lower prices and purchase drugs in bulk. Government bodies, or large health insurers, could use their bargaining power to influence drug prices. In recent years, there have also been calls for brand-name drug firms to voluntarily license their patent-protected products to the Medicines Patent Pool, a United Nations–backed organization that works to broaden access to medicines in low- and middle-income countries.^32,33,34^

Our results also showed differences in drug affordability across countries, reflecting variations in prices or minimum wages, or both. Even though countries in Southeast Asia generally had some of the lowest prices among the countries in our study, they also had some of the lowest levels of affordability due to low minimum wages. For instance, people in India had to work the most number of days at minimum wage, approximately 10 days, to pay for a monthly regimen of tenofovir disoproxil out of pocket. Given that we used minimum wage as the basis for our affordability estimates, these estimates represent affordability for the poorest in society and may be less representative in countries where there is greater income inequality. Even so, the results suggest important variability across low- and middle-income countries in terms of affordability.

### Limitations

This study had limitations. First, we relied on list prices reported by IQVIA, which excluded confidential discounts and rebates. However, list prices were the only data points that could be systematically compared between countries. This is an issue that affects all international comparisons of drug prices. Discounts are particularly common in high-income countries where drug purchasers exercise bargaining power; it is therefore likely that our study underestimated the difference in real affordability between richer and poorer countries.

Second, because minimum wage data were only available for 64 countries (plus Hong Kong), we were unable to measure affordability in all markets. Moreover, in some countries, the higher prevalence of informal labor markets and varying enforcement of minimum wage laws result in a substantial proportion of people earning less than the minimum wage. For the Central America and West Africa regions, we relied on data for a single country from each; this may have led us to overestimate or underestimate affordability in the regions.

Third, although the WHO Model List of Essential Medicines specifies formulations of active ingredients (eg, 20-mg simvastatin tablets), our price indices included data across all strength-form combinations of individual ingredients. For affordability calculations, we relied on price data for specific formulations.

Finally, our analysis was limited by data availability on products and countries, as well as by variations in the commercial channels tracked by IQVIA across countries, meaning the data may not have been nationally representative in all cases. We excluded certain disease areas, such as tuberculosis and malaria, due to insufficient data. The coverage of low-income countries, particularly in Africa, was limited. Additionally, IQVIA’s aggregation of data for West Africa and Central America restricted more detailed analysis.

## Conclusions

In this cross-sectional study, we observed significant variation in the prices and affordability of 549 essential medicines across 72 markets throughout the world in 2022. Many low- and middle-income countries paid higher prices for the same essential medicines compared to wealthier countries, placing a disproportionate cost burden on patients in poorer nations. There is a need for international strategies aimed at achieving more equitable outcomes.

## Notes

Supplement 1. eAppendix 1. Countries and medicines included in the analysis eAppendix 2. Definitions of drug categories eAppendix 3. Data used in the affordability calculations eAppendix 4. Descriptive statistics on spending by country eAppendix 5. Additional results tables and figure eAppendix 6. Main results without purchasing power parity adjustments

Supplement 2. Data Sharing Statement
