# Supplementary material for: Prices and Affordability of Essential Medicines in 72 Low-, Middle-, and High-Income Markets
Source: JAMA Health Forum. 2025 Aug 15;6(8):e252043. doi: 10.1001/jamahealthforum.2025.2043 (PMC12357183; doi:10.1001/jamahealthforum.2025.2043)
Supplement: Supplement 1. — eAppendix 1. Countries and medicines included in the analysis eAppendix 2. Definitions of drug categories eAppendix 3. Data used in the affordability calculations eAppendix 4. Descriptive statistics on spending by country eAppendix 5. Additional results tables and figure eAppendix 6. Main results without purchasing power parity adjustments [file jamahealthforum-e252043-s001.pdf]

Supplemental Online Content

Wouters OJ, Denolle C, Wei J, Papanicolas I. Prices and affordability of essential medicines in 72 low-, middle-, and high-income markets. *JAMA Health Forum*. 2025;6(8):e252043. doi:10.1001/jamadermatol.2025.2740

eAppendix 1. Countries and medicines included in the analysis
eAppendix 2. Definitions of drug categories
eAppendix 3. Data used in the affordability calculations
eAppendix 4. Descriptive statistics on spending by country
eAppendix 5. Additional results
eAppendix 6. Main results without purchasing power parity adjustments

This supplemental material has been provided by the authors to give readers additional information about their work.

© 2025 Wouters OJ et al. JAMA Health Forum.

eAppendix 1. Countries and medicines included in the analysis

Table A1. List of medicines included in the analysis.

Number	Drug name [a]	Drug category
1	Abiraterone	Cancer medicines
2	Acetazolamide	Other
3	Acetic Acid	Other
4	Acetohexamide	Diabetes Medication
5	Acetylsalicylic Acid	Cardiovascular disease drugs / Migraine and pain medications
6	Aciclovir	Other
7	Aclidinium	Asthma and COPD medicines
8	Adalimumab	Other
9	Afatinib	Cancer medicines
10	Allopurinol	Other
11	All-Trans Retinoic Acid	Cancer medicines
12	Alteplase	Cardiovascular disease drugs
13	Amcinonide	Other
14	Amikacin	Antibiotics
15	Amiloride	Cardiovascular disease drugs
16	Amiodarone	Cardiovascular disease drugs
17	Amitriptyline	Medicines for mental and behavioural disorders
18	Amlodipine	Cardiovascular disease drugs
19	Amlodipine / Benazepril	Cardiovascular disease drugs
20	Amlodipine / Celecoxib	Cardiovascular disease drugs
21	Amlodipine / Enalapril	Cardiovascular disease drugs
22	Amlodipine / Fimasartan	Cardiovascular disease drugs
23	Amlodipine / Irbesartan	Cardiovascular disease drugs
24	Amlodipine / Lisinopril	Cardiovascular disease drugs
25	Amlodipine / Losartan	Cardiovascular disease drugs
26	Amlodipine / Olmesartan Medoxomil	Cardiovascular disease drugs
27	Amlodipine / Perindopril	Cardiovascular disease drugs
28	Amlodipine / Ramipril	Cardiovascular disease drugs
29	Amlodipine / Telmisartan	Cardiovascular disease drugs
30	Amlodipine / Valsartan	Cardiovascular disease drugs
31	Amoxicillin	Antibiotics
32	Amoxicillin / Clavulanic Acid	Antibiotics
33	Amphotericin B	Other
34	Ampicillin	Antibiotics
35	Anastrozole	Cancer medicines
36	Anidulafungin	Other
37	Apixaban	Cardiovascular disease drugs
38	Aprepitant	Other
39	Arsenic Trioxide	Cancer medicines
40	Asparaginase	Cancer medicines

© 2025 Wouters OJ et al. JAMA Health Forum.

41	Atenolol	Cardiovascular disease drugs
42	Atorvastatin	Cardiovascular disease drugs
43	Atracurium	Other
44	Atropine	Other
45	Avibactam / Ceftazidime	Antibiotics
46	Azathioprine	Other
47	Azilsartan Medoxomil	Cardiovascular disease drugs
48	Azilsartan Medoxomil / Cilnidipine	Cardiovascular disease drugs
49	Azithromycin	Antibiotics
50	Barnidipine	Cardiovascular disease drugs
51	Beclometasone	Asthma and COPD medicines
52	Beclometasone / Formoterol	Asthma and COPD medicines
53	Benazepril	Cardiovascular disease drugs
54	Benazepril / Hydrochlorothiazide	Cardiovascular disease drugs
55	Bendamustine	Cancer medicines
56	Benidipine	Cardiovascular disease drugs
57	Benidipine / Telmisartan	Cardiovascular disease drugs
58	Benserazide / Levodopa	Other
59	Benzoyl Peroxide	Other
60	Benzyl Benzoate	Other
61	Benzylpenicillin	Antibiotics
62	Besifloxacin	Antibiotics
63	Betamethasone	Other
64	Betaxolol	Other
65	Bevacizumab	Other
66	Bicalutamide	Cancer medicines
67	Bifonazole	Other
68	Biperiden	Other
69	Bisacodyl	Other
70	Bisoprolol	Cardiovascular disease drugs
71	Bleomycin	Cancer medicines
72	Bortezomib	Cancer medicines
73	Budesonide	Asthma and COPD medicines
74	Budesonide / Formoterol	Asthma and COPD medicines
75	Budesonide / Salmeterol	Asthma and COPD medicines
76	Bumetanide	Cardiovascular disease drugs
77	Bupivacaine	Other
78	Buprenorphine	Medicines for mental and behavioural disorders
79	Bupropion	Medicines for mental and behavioural disorders
80	Calamine	Other
81	Calcipotriol	Other
82	Calcitriol	Other
83	Calcium Folinate	Cancer medicines
84	Canagliflozin	Diabetes Medication
85	Candesartan	Cardiovascular disease drugs
86	Capecitabine	Cancer medicines

© 2025 Wouters OJ et al. JAMA Health Forum.

87	Captopril	Cardiovascular disease drugs
88	Captopril / Hydrochlorothiazide	Cardiovascular disease drugs
89	Captopril / Indapamide	Cardiovascular disease drugs
90	Carbachol	Other
91	Carbamazepine	Medicines for mental and behavioural disorders
92	Carbetocin	Other
93	Carbidopa / Levodopa	Other
94	Carbimazole	Other
95	Carboplatin	Cancer medicines
96	Carteolol	Other
97	Carvedilol	Cardiovascular disease drugs
98	Caspofungin	Other
99	Cefalexin	Antibiotics
100	Cefazolin	Antibiotics
101	Cefiderocol	Antibiotics
102	Cefixime	Antibiotics
103	Cefotaxime	Antibiotics
104	Ceftazidime	Antibiotics
105	Ceftriaxone	Antibiotics
106	Cefuroxime	Antibiotics
107	Certolizumab Pegol	Other
108	Cetirizine	Other
109	Chlorambucil	Cancer medicines
110	Chloramphenicol	Antibiotics
111	Chlorhexidine	Other
112	Chloroprocaine	Other
113	Chloroquine	Other
114	Chlorothiazide	Cardiovascular disease drugs
115	Chlorpromazine	Medicines for mental and behavioural disorders
116	Chlorpropamide	Diabetes Medication
117	Chlortalidone	Cardiovascular disease drugs
118	Chlortetracycline	Antibiotics
119	Ciclesonide	Asthma and COPD medicines
120	Ciclosporin	Other
121	Cilastatin / Imipenem	Antibiotics
122	Cilazapril	Cardiovascular disease drugs
123	Cilazapril / Hydrochlorothiazide	Cardiovascular disease drugs
124	Cilnidipine	Cardiovascular disease drugs
125	Cilnidipine / Olmesartan Medoxomil	Cardiovascular disease drugs
126	Cilnidipine / Telmisartan	Cardiovascular disease drugs
127	Cilnidipine / Valsartan	Cardiovascular disease drugs
128	Cimetidine	Other
129	Cinchocaine	Other
130	Ciprofloxacin	Antibiotics
131	Cisplatin	Cancer medicines
132	Citalopram	Medicines for mental and behavioural disorders

© 2025 Wouters OJ et al. JAMA Health Forum.

133	Clarithromycin	Antibiotics
134	Clevidipine	Cardiovascular disease drugs
135	Clindamycin	Antibiotics
136	Clomifene	Other
137	Clomipramine	Medicines for mental and behavioural disorders
138	Clopidogrel	Cardiovascular disease drugs
139	Clotrimazole	Other
140	Cloxacillin	Antibiotics
141	Clozapine	Medicines for mental and behavioural disorders
142	Coal Tar	Other
143	Codeine	Migraine and pain medications
144	Colistin (Injection)	Antibiotics
145	Cyclizine	Other
146	Cyclopentolate	Other
147	Cyclophosphamide	Cancer medicines
148	Cytarabine	Cancer medicines
149	Dabigatran	Cardiovascular disease drugs
150	Dacarbazine	Cancer medicines
151	Daclatasvir	Hepatitis B and C medicines
152	Daclatasvir / Sofosbuvir	Hepatitis B and C medicines
153	Dactinomycin	Cancer medicines
154	Dalteparin	Cardiovascular disease drugs
155	Dapagliflozin	Diabetes Medication
156	Darbepoetin Alfa	Other
157	Dasabuvir	Hepatitis B and C medicines
158	Dasatinib	Cancer medicines
159	Daunorubicin	Cancer medicines
160	Deferasirox	Other
161	Deferoxamine	Other
162	Delapril	Cardiovascular disease drugs
163	Delapril / Indapamide	Cardiovascular disease drugs
164	Delapril / Manidipine	Cardiovascular disease drugs
165	Desmopressin	Cardiovascular disease drugs
166	Desoximetasone	Other
167	Dexamethasone	Cancer medicines
168	Dexlansoprazole	Other
169	Dexrabeprazole	Other
170	Diazepam	Medicines for mental and behavioural disorders
171	Diazoxide	Other
172	Dicloxacillin	Antibiotics
173	Diflorasone	Other
174	Diflucortolone	Other
175	Difluprednate	Other
176	Digoxin	Cardiovascular disease drugs
177	Docetaxel	Cancer medicines
178	Docusate Sodium	Other

© 2025 Wouters OJ et al. JAMA Health Forum.

179	Dolasetron	Other
180	Dopamine	Cardiovascular disease drugs
181	Doxorubicin	Cancer medicines
182	Doxycycline	Antibiotics
183	Eberconazole	Other
184	Econazole	Other
185	Edoxaban	Cardiovascular disease drugs
186	Efinaconazole	Other
187	Empagliflozin	Diabetes Medication
188	Enalapril	Cardiovascular disease drugs
189	Enalapril / Hydrochlorothiazide	Cardiovascular disease drugs
190	Enalapril / Indapamide	Cardiovascular disease drugs
191	Enalapril / Lercanidipine	Cardiovascular disease drugs
192	Enalapril / Nitrendipine	Cardiovascular disease drugs
193	Enoxaparin	Cardiovascular disease drugs
194	Entecavir	Hepatitis B and C medicines
195	Enzalutamide	Cancer medicines
196	Ephedrine	Other
197	Epinephrine	Cardiovascular disease drugs / Asthma and COPD medicines
198	Epinephrine / Lidocaine	Other
199	Epoetin Alfa	Other
200	Epoetin Beta	Other
201	Epoetin Theta	Other
202	Eprosartan	Cardiovascular disease drugs
203	Eprosartan / Hydrochlorothiazide	Cardiovascular disease drugs
204	Ergometrine	Other
205	Erlotinib	Cancer medicines
206	Erythromycin	Antibiotics
207	Escitalopram	Medicines for mental and behavioural disorders
208	Esomeprazole	Other
209	Estradiol Cypionate / Medroxyprogesterone Acetate	Other
210	Etanercept	Other
211	Ethinylestradiol / Etonogestrel	Other
212	Ethinylestradiol / Levonorgestrel	Other
213	Ethinylestradiol / Norethisterone	Other
214	Ethosuximide	Other
215	Etonogestrel-Releasing Implant	Other
216	Etoposide	Cancer medicines
217	Everolimus	Cancer medicines
218	Exemestane	Cancer medicines
219	Famotidine	Other
220	Felodipine	Cardiovascular disease drugs
221	Felodipine / Ramipril	Cardiovascular disease drugs
222	Fentanyl	Migraine and pain medications
223	Fenticonazole	Other

© 2025 Wouters OJ et al. JAMA Health Forum.

224	Ferrous Salt	Other
225	Ferrous Salt / Folic Acid	Other
226	Fexofenadine	Other
227	Filgrastim	Cancer medicines
228	Fimasartan	Cardiovascular disease drugs
229	Fimasartan / Hydrochlorothiazide	Cardiovascular disease drugs
230	Flucloxacillin	Antibiotics
231	Fluconazole	Other
232	Flucytosine	Other
233	Fludarabine	Cancer medicines
234	Fludrocortisone	Other
235	Fludroxycortide	Other
236	Flunisolide	Asthma and COPD medicines
237	Fluocinolone Acetonide	Other
238	Fluocinonide	Other
239	Fluocortolone	Other
240	Fluorouracil	Cancer medicines
241	Fluoxetine	Medicines for mental and behavioural disorders
242	Fluphenazine	Medicines for mental and behavioural disorders
243	Flutamide	Cancer medicines
244	Fluticasone	Asthma and COPD medicines
245	Fluticasone / Formoterol	Asthma and COPD medicines
246	Fluticasone Furoate / Vilanterol	Asthma and COPD medicines
247	Flutrimazole	Other
248	Fluvastatin	Cardiovascular disease drugs
249	Fluvoxamine	Medicines for mental and behavioural disorders
250	Folic Acid	Other
251	Formoterol / Mometasone	Asthma and COPD medicines
252	Fosfomycin (Injection)	Antibiotics
253	Fosinopril	Cardiovascular disease drugs
254	Fosinopril / Hydrochlorothiazide	Cardiovascular disease drugs
255	Furosemide	Cardiovascular disease drugs
256	Gatifloxacin	Antibiotics
257	Gefitinib	Cancer medicines
258	Gemcitabine	Cancer medicines
259	Gentamicin	Antibiotics
260	Glecaprevir / Pibrentasvir	Hepatitis B and C medicines
261	Glibenclamide	Diabetes Medication
262	Gliclazide	Diabetes Medication
263	Glimepiride	Diabetes Medication
264	Glipizide	Diabetes Medication
265	Gliquidone	Diabetes Medication
266	Glucagon	Other
267	Glyceryl Trinitrate	Cardiovascular disease drugs
268	Glycopyrronium	Asthma and COPD medicines
269	Golimumab	Other

© 2025 Wouters OJ et al. JAMA Health Forum.

270	Goserelin	Cancer medicines
271	Granisetron	Other
272	Griseofulvin	Other
273	Halometasone	Other
274	Haloperidol	Medicines for mental and behavioural disorders
275	Halothane	Other
276	Heparin Sodium	Cardiovascular disease drugs
277	Homatropine	Other
278	Hydralazine	Cardiovascular disease drugs
279	Hydrochlorothiazide	Cardiovascular disease drugs
280	Hydrochlorothiazide / Imidapril	Cardiovascular disease drugs
281	Hydrochlorothiazide / Irbesartan	Cardiovascular disease drugs
282	Hydrochlorothiazide / Lisinopril	Cardiovascular disease drugs
283	Hydrochlorothiazide / Losartan	Cardiovascular disease drugs
284	Hydrochlorothiazide / Moexipril	Cardiovascular disease drugs
285	Hydrochlorothiazide / Olmesartan Medoxomil	Cardiovascular disease drugs
286	Hydrochlorothiazide / Perindopril	Cardiovascular disease drugs
287	Hydrochlorothiazide / Quinapril	Cardiovascular disease drugs
288	Hydrochlorothiazide / Ramipril	Cardiovascular disease drugs
289	Hydrochlorothiazide / Telmisartan	Cardiovascular disease drugs
290	Hydrochlorothiazide / Valsartan	Cardiovascular disease drugs
291	Hydrochlorothiazide / Zofenopril	Cardiovascular disease drugs
292	Hydrocortisone	Cancer medicines
293	Hydromorphone	Migraine and pain medications
294	Hydroxocobalamin	Other
295	Hydroxycarbamide	Cancer medicines
296	Hydroxychloroquine	Other
297	Ibrutinib	Cancer medicines
298	Ibuprofen	Migraine and pain medications
299	Ifosfamide	Cancer medicines
300	Imatinib	Cancer medicines
301	Imidapril	Cardiovascular disease drugs
302	Indapamide	Cardiovascular disease drugs
303	Indapamide / Lisinopril	Cardiovascular disease drugs
304	Indapamide / Olmesartan Medoxomil	Cardiovascular disease drugs
305	Indapamide / Perindopril	Cardiovascular disease drugs
306	Indapamide / Ramipril	Cardiovascular disease drugs
307	Indapamide / Telmisartan	Cardiovascular disease drugs
308	Indapamide / Valsartan	Cardiovascular disease drugs
309	Indometacin	Other
310	Infliximab	Other
311	Insulin Degludec	Diabetes Medication
312	Insulin Detemir	Diabetes Medication
313	Insulin Glargine	Diabetes Medication
314	Insulin Injection (Soluble)	Diabetes Medication

© 2025 Wouters OJ et al. JAMA Health Forum.

315	Intermediate-Acting Insulin	Diabetes Medication
316	Ipratropium Bromide	Asthma and COPD medicines
317	Irbesartan	Cardiovascular disease drugs
318	Irbesartan / Levamlodipine	Cardiovascular disease drugs
319	Irinotecan	Cancer medicines
320	Isoconazole	Other
321	Isoflurane	Other
322	Isosorbide Dinitrate	Cardiovascular disease drugs
323	Isradipine	Cardiovascular disease drugs
324	Itraconazole	Other
325	Kanamycin	Antibiotics
326	Ketamine	Other
327	Ketoconazole	Other
328	Lacidipine	Cardiovascular disease drugs
329	Lactulose	Other
330	Lafutidine	Other
331	Lamotrigine	Other
332	Lanoconazole	Other
333	Lansoprazole	Other
334	Latanoprost	Other
335	Ledipasvir / Sofosbuvir	Hepatitis B and C medicines
336	Lenalidomide	Cancer medicines
337	Lercanidipine	Cardiovascular disease drugs
338	Lercanidipine / Valsartan	Cardiovascular disease drugs
339	Letrozole	Cancer medicines
340	Leuprorelin	Cancer medicines
341	Levamlodipine	Cardiovascular disease drugs
342	Levamlodipine / Losartan	Cardiovascular disease drugs
343	Levamlodipine / Olmesartan Medoxomil	Cardiovascular disease drugs
344	Levamlodipine / Telmisartan	Cardiovascular disease drugs
345	Levamlodipine / Valsartan	Cardiovascular disease drugs
346	Levobunolol	Other
347	Levofloxacin	Antibiotics
348	Levonorgestrel	Other
349	Levothyroxine	Other
350	Lidocaine	Cardiovascular disease drugs
351	Linezolid	Antibiotics
352	Lisinopril	Cardiovascular disease drugs
353	Lithium Carbonate	Medicines for mental and behavioural disorders
354	Lomefloxacin	Antibiotics
355	Loperamide	Other
356	Loratadine	Other
357	Lorazepam	Other
358	Losartan	Cardiovascular disease drugs
359	Lovastatin	Cardiovascular disease drugs
360	Luliconazole	Other

© 2025 Wouters OJ et al. JAMA Health Forum.

361	Magnesium Sulfate	Other
362	Manidipine	Cardiovascular disease drugs
363	Mannitol	Cardiovascular disease drugs
364	Medroxyprogesterone Acetate	Other
365	Melphalan	Cancer medicines
366	Mercaptopurine	Cancer medicines
367	Meropenem	Antibiotics
368	Meropenem / Vaborbactam	Antibiotics
369	Mesalazine	Other
370	Mesna	Other
371	Metformin	Diabetes Medication
372	Methadone	Medicines for mental and behavioural disorders / Migraine and pain medications
373	Methimazole	Other
374	Methotrexate	Cancer medicines
375	Methoxy Polyethylene Glycol-Epoetin Beta	Other
376	Methyldopa	Cardiovascular disease drugs
377	Methylergometrine	Other
378	Methylprednisolone	Cancer medicines
379	Methylprednisolone Aceponate	Other
380	Metoclopramide	Other
381	Metoprolol	Cardiovascular disease drugs
382	Metronidazole	Antibiotics
383	Micafungin	Other
384	Miconazole	Other
385	Midazolam	Other
386	Mifepristone / Misoprostol	Other
387	Misoprostol	Other
388	Moexipril	Cardiovascular disease drugs
389	Mometasone	Asthma and COPD medicines
390	Morphine	Migraine and pain medications
391	Moxifloxacin	Antibiotics
392	Mupirocin	Other
393	Nadroparin	Cardiovascular disease drugs
394	Nafcillin	Antibiotics
395	Natamycin	Antibiotics
396	Neostigmine	Other
397	Neticonazole	Other
398	Netilmicin	Antibiotics
399	Nicardipine	Cardiovascular disease drugs
400	Nicotine Replacement Therapy	Medicines for mental and behavioural disorders
401	Nifedipine	Other
402	Nilotinib	Cancer medicines
403	Nilutamide	Cancer medicines
404	Nilvadipine	Cardiovascular disease drugs
405	Nimodipine	Cardiovascular disease drugs

© 2025 Wouters OJ et al. JAMA Health Forum.

406	Nisoldipine	Cardiovascular disease drugs
407	Nitrendipine	Cardiovascular disease drugs
408	Nitrofurantoin	Antibiotics
409	Nitrous Oxide	Other
410	Nivolumab	Cancer medicines
411	Nizatidine	Other
412	Norethisterone	Other
413	Norfloxacin	Antibiotics
414	Nystatin	Other
415	Ofloxacin	Antibiotics
416	Olmesartan Medoxomil	Cardiovascular disease drugs
417	Ombitasvir / Paritaprevir / Ritonavir	Hepatitis B and C medicines
418	Omeprazole	Other
419	Omoconazole	Other
420	Ondansetron	Other
421	Oseltamivir	Other
422	Oxacillin	Antibiotics
423	Oxaliplatin	Cancer medicines
424	Oxiconazole	Other
425	Oxybuprocaine	Other
426	Oxycodone	Migraine and pain medications
427	Oxytetracycline	Antibiotics
428	Oxytocin	Other
429	Paclitaxel	Cancer medicines
430	Paliperidone	Medicines for mental and behavioural disorders
431	Palonosetron	Other
432	Pancreatic Enzymes	Other
433	Pantoprazole	Other
434	Paracetamol	Migraine and pain medications
435	Paroxetine	Medicines for mental and behavioural disorders
436	Pegaspargase	Cancer medicines
437	Pegylated Interferon Alfa (2A)	Hepatitis B and C medicines
438	Pegylated Interferon Alfa (2B)	Hepatitis B and C medicines
439	Pembrolizumab	Cancer medicines
440	Penicillamine	Other
441	Perindopril	Cardiovascular disease drugs
442	Permethrin	Other
443	Phenobarbital	Other
444	Phenoxymethylpenicillin	Antibiotics
445	Phenytoin	Other
446	Phytomenadione	Cardiovascular disease drugs
447	Pilocarpine	Other
448	Piperacillin / Tazobactam	Antibiotics
449	Plazomicin	Antibiotics
450	Podophyllotoxin	Other
451	Podophyllum Resin	Other

© 2025 Wouters OJ et al. JAMA Health Forum.

452	Polymyxin B (Injection)	Antibiotics
453	Potassium Iodide	Other
454	Potassium Permanganate	Other
455	Pravastatin	Cardiovascular disease drugs
456	Prednicarbate	Other
457	Prednisolone	Cancer medicines
458	Prednisone	Cancer medicines
459	Procaine	Other
460	Procarbazine	Cancer medicines
461	Propofol	Other
462	Propranolol	Migraine and pain medications
463	Propylthiouracil	Other
464	Prostaglandin E1	Other
465	Prostaglandin E2	Other
466	Protamine Sulfate	Cardiovascular disease drugs
467	Proxymetacaine	Other
468	Pyridostigmine	Other
469	Quinapril	Cardiovascular disease drugs
470	Rabeprazole	Other
471	Ramipril	Cardiovascular disease drugs
472	Ranitidine	Other
473	Ranitidine Bismuth Citrate	Other
474	Rasburicase	Other
475	Ribavirin	Hepatitis B and C medicines
476	Risperidone	Medicines for mental and behavioural disorders
477	Rituximab	Cancer medicines
478	Rivaroxaban	Cardiovascular disease drugs
479	Roxatidine	Other
480	Salbutamol	Asthma and COPD medicines
481	Salicylic Acid	Other
482	Senna	Other
483	Sertaconazole	Other
484	Sertraline	Medicines for mental and behavioural disorders
485	Silver Sulfadiazine	Other
486	Simvastatin	Cardiovascular disease drugs
487	Sodium Nitroprusside	Cardiovascular disease drugs
488	Sofosbuvir	Hepatitis B and C medicines
489	Sofosbuvir / Velpatasvir	Hepatitis B and C medicines
490	Spectinomycin	Antibiotics
491	Spirapril	Cardiovascular disease drugs
492	Spironolactone	Cardiovascular disease drugs
493	Streptokinase	Cardiovascular disease drugs
494	Sulconazole	Other
495	Sulfamethoxazole / Trimethoprim	Antibiotics
496	Sulfasalazine	Other
497	Sumatriptan	Migraine and pain medications

© 2025 Wouters OJ et al. JAMA Health Forum.

498	Suxamethonium	Other
499	Tacalcitol	Other
500	Tacrolimus	Other
501	Tamoxifen	Cancer medicines
502	Tegoprazan	Other
503	Telmisartan	Cardiovascular disease drugs
504	Temocapril	Cardiovascular disease drugs
505	Tenofovir Disoproxil Fumarate	Hepatitis B and C medicines
506	Terbinafine	Other
507	Terbutaline	Asthma and COPD medicines
508	Testosterone	Other
509	Tetracaine	Other
510	Tetracycline	Antibiotics
511	Thalidomide	Cancer medicines
512	Thiopental	Other
513	Tiabendazole	Other
514	Timolol	Other
515	Tioconazole	Other
516	Tioguanine	Cancer medicines
517	Tiotropium Bromide	Asthma and COPD medicines
518	Tobramycin	Antibiotics
519	Tolbutamide	Diabetes Medication
520	Torasemide	Cardiovascular disease drugs
521	Tosufloxacin	Antibiotics
522	Trandolapril	Cardiovascular disease drugs
523	Tranexamic Acid	Cardiovascular disease drugs
524	Trastuzumab	Cancer medicines
525	Trihexyphenidyl	Other
526	Trimethoprim	Antibiotics
527	Triptorelin	Cancer medicines
528	Tropisetron	Other
529	Ulipristal	Other
530	Ulobetasol	Other
531	Umeclidinium	Asthma and COPD medicines
532	Urea	Other
533	Valaciclovir	Other
534	Valganciclovir	Other
535	Valproic Acid (Sodium Valproate)	Medicines for mental and behavioural disorders
536	Valsartan	Cardiovascular disease drugs
537	Vancomycin	Antibiotics
538	Varenicline	Medicines for mental and behavioural disorders
539	Vecuronium	Other
540	Verapamil	Cardiovascular disease drugs
541	Vinblastine	Cancer medicines
542	Vincristine	Cancer medicines
543	Vinorelbine	Cancer medicines

© 2025 Wouters OJ et al. JAMA Health Forum.

544	Vonoprazan	Other
545	Voriconazole	Other
546	Warfarin	Cardiovascular disease drugs
547	Xylometazoline	Other
548	Zofenopril	Cardiovascular disease drugs
549	Zoledronic Acid	Other

Abbreviations: COPD, chronic obstructive pulmonary disease.

[a] Medicine names may be spelt differently (or medicines may go by different names) across countries. For example, paracetamol is called acetaminophen in some countries, and salbutamol is called albuterol in the United States.

© 2025 Wouters OJ et al. JAMA Health Forum.

Table A2. Countries included in the IQVIA dataset, by WHO region.[a]

Americas (AMRO)	Europe (EURO)	Africa (AFRO)	Western Pacific (WPRO)	South-East Asia (SEARO)	Eastern Mediterranean (EMRO)
Argentina	Austria	Benin	Australia	Bangladesh	Egypt
Brazil	Belarus	Burkina Faso	China [b]	India	Jordan
Canada	Belgium	Cameroon	Japan	Indonesia	Kuwait
Chile	Bosnia	Chad	Malaysia	Sri Lanka	Lebanon
Colombia	Bulgaria	Congo (Rep. of)	New Zealand	Thailand	Morocco
Costa Rica	Croatia	Gabon	Philippines		Pakistan
Dominican Rep	Czech Republic	Guinea	Singapore		Saudi Arabia
Ecuador	Estonia	Côte d'Ivoire	South Korea		Tunisia
El Salvador	Finland	Mali	Taiwan		UAE
Guatemala	France	Niger	Vietnam		
Honduras	Germany	Senegal			
Mexico	Greece	South Africa			
Nicaragua	Hungary	Togo			
Panama	Ireland				
Peru	Italy				
Uruguay	Kazakhstan				
USA	Latvia				
	Lithuania				
	Luxembourg				
	Netherlands				
	Norway				
	Poland				
	Portugal				
	Romania				
	Russia				
	Serbia				
	Slovakia				

© 2025 Wouters OJ et al. JAMA Health Forum.

	Slovenia				
	Spain				
	Sweden				
	Switzerland				
	Türkiye				
	UK				

Rep., Republic (of the Congo); UAE, United Arab Emirates; UK, United Kingdom; USA, United States of America.

[a] Data were available for 69 individual countries, plus Hong Kong; data for an additional 6 countries were aggregated as Central America, and data for a further 12 countries were aggregated as West Africa. The sample thus consisted of 72 markets, covering 87 countries (69 individual countries and 2 regions of 18 countries) plus the special administrative region of Hong Kong.

[b] Data for Hong Kong reported separately.

© 2025 Wouters OJ et al. JAMA Health Forum.

eAppendix 2. Definitions of drug categories

Table A3. Essential medicines included in each drug category (by section on the World Health Organization EML).

**Drug category**	**EML category**
Overall sample	All drugs
Antibiotics	6.2.1, 6.2.2, 6.2.3, and 21.1 (excl. aciclovir)
Anti-diabetics	18.5
Cancer medicines	8.2.1 to 8.2.4
Cardiovascular disease drugs (includes certain anticoagulants and diuretics)	10.2, 12, and 16
Hepatitis B/C medicines	6.4.4
COPD / asthma medicines	25
Medicines for mental and behavioural disorders	24
Pain medicines	2.1, 2.2
Anti-migraine medicines	7.1, 7.2

EML indicates Essential Medicines List.

© 2025 Wouters OJ et al. JAMA Health Forum.

eAppendix 3. Data used in the affordability calculations

Table A4. Data on national minimum wages.

Country	Minimum monthly wage (US$) [a]
Argentina	443.3
Australia	2442.0
Bangladesh	87.2
Belarus	183.7
Belgium	1940.0
Bosnia	292.0
Brazil	234.7
Bulgaria	381.7
Canada	1897.7
Chile	458.0
China	286.5
Colombia	235.0
Costa Rica [b]	501.5
Côte d'Ivoire [b]	96.2
Croatia	655.1
Czech Republic	693.6
Dominican Republic	294.9
Ecuador	425.0
Egypt	156.6
Estonia	688.7
France	1732.9
Germany	1831.3
Greece	876.0
Hong Kong	829.4
Hungary	536.8
India	58.9
Indonesia	180.5
Ireland	1868.6
Japan	1266.7
Jordan	366.2
Kazakhstan	130.4
Kuwait	244.9
Latvia	526.5
Lebanon	1724.7
Lithuania	768.7
Luxembourg	2436.1
Malaysia	340.8
Mexico	223.3
Morocco	285.6
Netherlands	1849.4

© 2025 Wouters OJ et al. JAMA Health Forum.

New Zealand	2329.9
Pakistan	122.0
Peru	267.3
Philippines	148.0
Poland	675.2
Portugal	866.1
Romania	543.9
Russia	173.7
Saudi Arabia	1066.7
Serbia	422.6
Slovakia	680.3
Slovenia	1131.4
South Africa	248.1
South Korea	1482.4
Spain	1228.6
Sri Lanka	62.9
Switzerland	4221.0
Taiwan	846.9
Thailand	248.4
Tunisia	125.5
Türkiye	391.0
UK	1940.4
Uruguay	470.3
USA	1256.7
Vietnam	167.4

Source: International Labour Organization.
UK, United Kingdom; USA, United States of America.

[a] All amounts reported in 2022 US dollars, Russia and Sri Lanka (2021).
[b] Wage data from Costa Rica were used to estimate affordability for Central America (6-country group); data from Côte d'Ivoire were used to estimate affordability for West Africa (12-country group).

© 2025 Wouters OJ et al. JAMA Health Forum.

Table A5. Treatment regimens used in the affordability calculations.

Essential medicine	Drug category	Treatment regimen (per month [30 days])	Source
Amoxicillin	Antibiotics	500mg 3x per day; for 7 days (21 tablets)	https://www.who.int/medicines/areas/access/OMS_Medicine_prices.pdf
Escitalopram	Mental and behavioural disorders	10mg once daily x 30 days (30 tablets)	https://www.who.int/medicines/areas/access/OMS_Medicine_prices.pdf
Ibuprofen	Migraine and pain	400mg 3x per day; for 7 days (21 tablets)	https://www.nhs.uk/medicines/ibuprofen-for-adults/how-and-when-to-take-ibuprofen/<br><br>https://www.mayoclinic.org/drugs-supplements/ibuprofen-oral-route/proper-use/drg-20070602
Insulin injection (soluble)	Diabetes	1 vial of 300 units per month	https://www.who.int/ncds/management/2.2_DIABETES_Type_2_management-WHOPEN.pdf
Losartan	Cardiovascular disease	50mg once daily x 30 days (30 tablets)	https://www.who.int/medicines/areas/access/OMS_Medicine_prices.pdf
Paclitaxel	Cancer	300mg vial (1 pack)	https://www.ncbi.nlm.nih.gov/pmc/articles/PMC8259378/#!po=37.5000
Salbutamol	Asthma and chronic obstructive pulmonary disease	0.1mg/dose inhaler; 2 doses daily x 30 days<br><br>An average of 2 puffs per day was assumed for comparison across countries.	https://www.nhs.uk/medicines/salbutamol-inhaler/how-and-when-to-use-salbutamol-inhalers/
Tenofovir disoproxil	Hepatitis B and C	300mg tenofovir disoproxil fumarate once daily (which is equivalent to 245 mg tenofovir disoproxil) x 30 days (30 tablets)	https://bnf.nice.org.uk/drug/ribavirin.html (for strength)<br><br>https://www.who.int/publications/i/item/9789241550345)

© 2025 Wouters OJ et al. JAMA Health Forum.

eAppendix 4. Descriptive statistics on spending by country

Table A6. Spending on and utilization of essential medicines by country, 2022.

Country	N	Total spending on essential medicines (US$, billions)	Spending per capita on essential medicines (US$)	Number of doses consumed per capita	% Generic market share (by volume)	% Generic market share (by value)	GDP per capita (US$)	Health expenditure per capita (US$)	Health expenditure (% of GDP)
Argentina	353	4.6	99.1	289.2	76.7	71.8	13,650.6	1,044.8	9.7
Australia	389	5.9	226.7	989.2	64.8	29.2	65,077.7	7,055.4	10.5
Austria	381	5.6	624.8	546.5	65.3	28.8	52,084.7	6,505.0	12.1
Bangladesh	308	1.6	9.3	178.8	97.5	96.4	2,687.9	57.9	2.4
Belarus	333	0.4	43.3	658.0	50.9	67.4	7,994.6	468.0	6.6
Belgium	383	4.8	408.3	718.0	68.0	23.4	49,942.1	5,680.2	11.0
Bosnia	289	0.3	78.0	527.9	63.1	48.8	7,587.6	692.0	9.6
Brazil	394	14.5	67.2	329.3	70.2	69.5	9,065.5	761.3	9.9
Bulgaria	345	1.0	158.0	642.2	53.4	30.5	13,974.4	1,040.0	8.6
Canada	392	15.0	385.2	721.0	62.0	28.6	55,509.4	6,470.1	12.3
Central America	358	1.2	229.8	676.0	68.1	58.5	.	.	.
Chile	317	0.8	42.1	200.5	68.5	61.5	15,411.2	1,518.0	9.3
China	416	43.0	30.4	98.2	68.1	58.7	12,662.6	670.5	5.4
Colombia	340	0.8	15.6	147.9	48.5	60.0	6,657.1	557.5	9.0
Croatia	325	0.8	206.5	669.9	51.6	32.0	18,673.2	1,384.0	8.1
Czech Republic	374	2.0	190.1	649.5	65.1	29.0	27,226.6	2,498.5	9.5
Dominican Republic	325	0.5	45.7	112.0	77.7	78.1	10,111.2	416.9	4.9
Ecuador	320	0.7	41.4	194.7	75.8	70.9	6,476.6	494.3	8.3
Egypt	376	3.3	30.1	386.4	71.1	54.4	4,295.4	179.7	4.6
Estonia	325	0.2	126.7	504.8	54.4	32.1	28,114.1	2,094.5	7.5
Finland	347	2.0	356.4	804.1	63.6	27.3	50,734.7	5,488.0	10.2

© 2025 Wouters OJ et al. JAMA Health Forum.

France	416	18.1	266.8	783.7	83.4	37.5	40,886.3	5,380.9	12.3
Germany	438	25.4	303.2	745.5	68.2	29.2	48,718.0	6,626.0	12.9
Greece	367	2.0	191.6	768.1	50.0	33.5	20,867.3	1,845.8	9.2
Hong Kong	381	1.0	138.8	787.9	46.9	19.5	48,828.1	.	.
Hungary	369	1.3	138.8	605.5	73.7	35.3	18,355.8	1,382.0	7.4
India	412	9.9	7.0	113.8	78.2	80.4	2,366.3	74.0	3.3
Indonesia	345	1.8	6.6	43.9	82.1	70.2	4,787.9	160.6	3.7
Ireland	361	2.1	405.6	920.7	55.3	25.0	103,983.3	6,764.3	6.7
Italy	435	26.0	440.8	596.9	64.4	27.3	35,068.8	3,350.0	9.4
Japan	387	24.1	192.4	494.3	70.8	26.4	34,017.3	4,347.0	10.8
Jordan	266	0.2	17.6	114.1	74.8	59.6	4,311.0	299.1	7.3
Kazakhstan	331	0.7	33.3	322.3	61.1	56.3	11,484.4	403.0	3.9
Kuwait	225	0.2	38.5	163.2	63.2	41.4	42,823.8	1,860.8	5.8
Latvia	356	0.2	101.0	453.8	54.6	34.4	21,508.4	1,898.0	9.0
Lebanon	323	0.3	49.3	188.2	75.0	62.7	3,823.9	307.1	10.1
Lithuania	369	0.4	140.3	520.1	52.5	26.9	25,078.7	1,859.0	7.8
Luxembourg	286	0.2	235.5	546.2	42.9	17.9	125,006.0	7,636.0	5.7
Malaysia	360	1.3	37.4	334.8	35.6	25.1	11,993.2	487.0	4.4
Mexico	379	4.0	31.7	87.5	63.6	52.5	11,476.7	610.6	6.1
Morocco	287	1.1	28.5	175.2	69.3	64.2	3,442.0	221.1	5.7
Netherlands	335	1.9	107.7	548.1	85.2	50.5	57,025.0	6,539.0	11.3
New Zealand	341	0.9	166.6	1,160.9	74.3	27.6	48,216.5	4,906.1	10.0
Norway	390	2.2	396.7	833.4	63.1	32.7	108,798.5	9,163.0	10.1
Pakistan	330	1.7	7.2	274.9	64.9	76.3	1,589.3	43.1	2.9
Peru	308	0.3	9.8	96.4	64.2	63.8	7,239.1	412.2	6.2
Philippines	339	1.9	16.3	79.8	59.8	56.1	3,499.1	203.0	5.9
Poland	393	4.6	124.1	634.1	57.7	34.6	18,732.5	1,159.0	6.4
Portugal	403	2.0	195.2	717.9	70.1	38.6	24,515.3	2,747.0	11.1
Romania	348	2.8	147.5	554.3	46.5	26.0	15,692.3	963.0	6.5
Russia	409	9.8	68.1	564.7	45.2	38.0	15,445.2	936.0	7.4

© 2025 Wouters OJ et al. JAMA Health Forum.

Saudi Arabia	355	4.6	126.0	447.6	78.7	51.1	30,447.9	1,442.0	6.0
Serbia	317	0.7	103.1	753.1	70.8	44.9	9,537.7	919.2	10.0
Singapore	355	0.5	96.3	288.7	32.6	14.1	88,428.7	3,969.9	5.6
Slovakia	362	0.8	156.0	606.9	68.2	33.5	21,279.5	1,685.0	7.8
Slovenia	344	0.4	201.8	605.1	54.4	29.8	28,439.3	2,775.0	9.5
South Africa	376	1.8	30.2	366.6	74.4	56.7	6,766.5	583.7	8.3
South Korea	407	6.3	122.1	435.3	48.7	41.7	32,394.7	3,260.4	9.3
Spain	420	15.6	326.2	808.5	75.1	32.1	29,674.5	3,234.3	10.7
Sri Lanka	238	0.2	7.0	142.6	90.5	83.8	3,342.7	166.0	4.1
Sweden	342	2.3	222.3	884.6	72.1	28.6	56,299.5	6,901.0	11.2
Switzerland	371	5.2	587.2	588.0	63.4	32.1	93,259.9	10,897.5	11.8
Taiwan	398	3.4	144.9	370.6	54.8	22.4	32,609.6	.	.
Thailand	377	3.1	43.7	425.7	69.3	50.0	6,913.1	364.4	5.2
Tunisia	324	0.6	45.1	438.6	76.3	61.4	3,607.9	265.5	7.0
Türkiye	379	3.4	39.9	556.7	71.5	48.6	10,674.5	441.0	4.6
UAE	317	1.2	125.7	332.6	70.2	41.8	53,708.0	2,351.8	5.3
UK	422	20.5	302.4	895.5	75.4	35.1	45,564.2	5,738.5	12.4
Uruguay	317	0.2	61.3	337.1	79.7	72.8	20,499.2	1,620.3	9.4
USA	428	293.0	879.2	663.9	77.2	17.4	77,246.7	12,012.2	17.4
Vietnam	365	1.8	18.5	164.6	75.0	54.3	4,179.0	172.6	4.6
West Africa	341	1.1	39.6	437.7	86.4	77.6	.	.	.

Abbreviations: GDP, gross domestic product; UAE, United Arab Emirates; UK, United Kingdom; USA, United States of America.

Sources: All data from IQVIA (based on retail sales), except GDP per capita and health expenditure (World Bank). All data are from 2022, except health expenditure (2021) and health expenditure per capita (2021).

© 2025 Wouters OJ et al. JAMA Health Forum.

eAppendix 5. Additional results

Table A7. Price index results broken down by disease area, adjusted for purchasing power parity.

Country	WHO region	Full sample (N=549)	Antibiotics (N=58)	Asthma and COPD (N=20)	Cancer (N=68)	CVD (N=143)	Diabetes (N=17)	Hepatitis B and C (N=13)	Migraine and pain (N=11)	Mental health (N=23)
Lebanon	EMRO	18.1	51.6	9.4	15.2	26.2	12.6	5.8	9.3	22.3
Australia	WPRO	52.9	58.0	48.7	36.8	66.6	38.4	54.8	49.0	67.1
New Zealand	WPRO	65.4	80.5	35.0	56.0	64.3	47.1	56.6	21.3	33.5
Bangladesh	SEARO	77.7	134.6	36.0	44.2	92.0	59.9	28.5	22.1	59.2
Turkey	EURO	80.7	79.8	43.0	63.0	93.4	39.6	47.7	41.9	86.3
Sweden	EURO	84.5	159.2	59.9	61.0	73.2	54.6	62.9	48.4	56.0
Japan	WPRO	89.5	94.6	74.2	60.7	104.6	68.4	67.6	99.1	188.8
France	EURO	96.1	186.6	64.1	83.1	112.5	64.3	82.8	89.8	91.6
Netherlands	EURO	96.8	257.2	50.7	120.3	83.0	52.2	48.9	84.9	100.4
Pakistan	EMRO	97.6	149.4	43.7	160.5	119.0	40.2	16.8	25.6	80.1
Germany	EURO	100.0	100.0	100.0	100.0	100.0	100.0	100.0	100.0	100.0
South Korea	WPRO	103.4	105.8	56.5	52.3	140.3	47.3	27.8	61.8	91.3
Canada	AMRO	104.3	239.4	102.6	93.6	105.7	86.1	95.8	60.0	107.1
Finland	EURO	112.5	344.0	76.7	107.7	93.9	65.9	120.6	106.4	99.4
Czech Republic	EURO	120.5	165.7	70.1	131.7	103.7	101.2	110.9	98.3	115.1
Estonia	EURO	120.7	425.4	78.8	60.8	106.7	73.9	54.2	122.1	90.5
Lithuania	EURO	121.1	148.4	70.9	97.7	111.0	82.2	30.7	146.3	123.9
Portugal	EURO	124.1	91.0	78.2	77.6	141.4	86.3	22.5	121.6	128.4
UK	EURO	126.5	260.5	68.1	127.1	122.0	68.7	128.5	47.1	95.4
Singapore	WPRO	126.9	257.9	100.6	96.5	129.8	49.4	17.6	76.0	305.9
Norway	EURO	128.8	261.6	73.0	114.2	90.0	61.7	109.9	75.3	83.2

© 2025 Wouters OJ et al. JAMA Health Forum.

Serbia	EURO	129.9	104.9	100.3	123.0	125.6	79.0	64.7	94.7	91.7
Slovakia	EURO	130.4	171.6	72.4	110.8	115.1	95.8	102.6	105.0	70.1
Hong Kong	WPRO	132.1	169.5	59.9	80.2	127.8	59.8	21.7	36.9	112.1
Poland	EURO	133.1	125.9	87.7	107.3	172.9	101.0	88.8	121.2	130.7
Russia	EURO	133.5	187.3	88.8	106.1	165.0	109.3	25.5	80.9	275.8
China	WPRO	133.6	548.3	130.2	84.8	144.5	70.2	5.3	149.8	281.7
Latvia	EURO	138.7	216.5	89.8	101.7	111.5	90.5	17.5	129.4	106.5
India	SEARO	139.2	146.1	49.5	90.2	174.2	87.6	18.2	35.6	131.8
Luxembourg	EURO	139.5	153.4	86.4	100.3	167.5	74.4	96.6	116.1	116.4
Hungary	EURO	141.0	136.6	93.7	123.0	118.5	94.0	100.0	158.7	88.1
Greece	EURO	143.7	181.2	110.9	96.9	193.4	93.1	136.1	139.5	157.8
Bulgaria	EURO	144.3	157.2	102.2	118.2	172.9	114.7	122.8	136.9	129.4
South Africa	AFRO	150.8	120.2	84.2	100.1	212.4	59.7	21.1	77.6	123.0
Uruguay	AMRO	154.2	117.8	130.7	106.5	118.9	91.4	17.9	135.0	95.8
Ireland	EURO	156.2	225.3	86.4	134.3	136.5	82.4	95.3	113.3	129.9
Belarus	EURO	157.0	115.9	124.3	85.6	183.2	151.3	17.9	87.8	206.4
Spain	EURO	158.8	176.8	81.4	163.1	160.8	90.9	148.9	83.1	127.3
Romania	EURO	162.2	215.6	86.3	172.7	174.4	124.8	145.1	185.6	141.6
Switzerland	EURO	162.7	467.5	120.7	136.4	185.7	85.0	131.9	135.3	201.0
Sri Lanka	SEARO	165.3	425.0	48.0	90.4	244.6	65.7	.	109.9	118.2
Belgium	EURO	170.8	156.0	89.4	160.6	159.7	89.2	113.3	95.4	126.9
Vietnam	WPRO	172.2	148.7	104.1	118.2	226.5	110.6	9.6	61.7	198.9
Kazakhstan	EURO	174.5	197.4	158.3	153.2	180.2	113.1	15.7	76.2	264.8
Croatia	EURO	177.2	268.9	102.0	212.5	173.6	108.1	163.9	125.9	124.1
Taiwan	WPRO	180.3	136.2	71.0	113.4	173.7	119.2	45.0	90.4	129.9
Tunisia	EMRO	188.3	198.7	96.9	182.2	263.3	118.0	3.7	82.5	222.5
Slovenia	EURO	189.6	315.4	83.5	137.6	109.6	68.1	90.7	105.2	86.9
Bosnia	EURO	191.1	137.4	148.0	123.0	172.6	134.8	56.9	125.3	101.2
Saudi Arabia	EMRO	195.1	245.3	109.1	140.8	225.5	95.4	133.3	85.0	376.2

© 2025 Wouters OJ et al. JAMA Health Forum.

Malaysia	WPRO	195.4	322.7	78.0	134.2	269.7	91.3	36.4	88.7	441.6
Kuwait	EMRO	201.1	317.6	103.4	100.5	291.5	89.3	.	67.3	263.9
Peru	AMRO	208.0	153.3	97.7	152.2	266.2	162.7	28.1	142.5	391.2
Egypt	EMRO	208.4	257.2	106.7	202.1	226.3	88.4	23.3	63.4	174.1
Austria	EURO	222.5	676.6	137.6	237.0	211.5	128.1	162.7	121.7	220.2
Jordan	EMRO	224.9	399.2	94.2	197.1	283.0	118.0	.	116.3	187.3
Morocco	EMRO	238.4	278.3	100.5	258.7	298.8	130.7	19.0	149.5	249.8
Italy	EURO	242.4	273.0	105.1	223.9	197.3	125.7	193.5	181.0	189.7
UAE	EMRO	262.1	293.6	142.1	182.2	319.2	118.3	162.4	117.6	403.6
Chile	AMRO	271.4	642.7	119.5	168.6	394.9	198.0	123.7	224.3	383.0
USA	AMRO	298.2	110.7	468.0	184.0	342.7	472.6	65.7	43.0	102.1
West Africa [a]	AFRO	304.9	308.4	159.0	137.1	358.5	145.5	7.7	140.5	268.8
Ecuador	AMRO	309.3	294.5	97.9	210.3	414.4	175.5	.	196.6	564.6
Brazil	AMRO	313.5	557.5	100.6	298.8	287.5	111.0	118.2	319.9	316.0
Colombia	AMRO	316.0	255.6	151.1	176.1	363.2	171.3	121.4	219.5	496.5
Philippines	WPRO	321.5	462.5	155.9	210.9	353.6	122.4	30.0	163.1	564.1
Central America [a]	AMRO	385.4	449.3	209.1	246.2	540.8	193.1	9.6	249.1	577.3
Indonesia	SEARO	393.2	242.8	318.3	208.6	380.0	150.5	50.7	269.0	237.7
Thailand	SEARO	399.8	290.2	175.6	288.8	548.9	218.1	37.7	54.6	259.8
Dominican Republic	AMRO	412.1	627.4	220.5	179.2	508.0	193.4	3.4	298.5	527.4
Mexico	AMRO	432.1	469.8	196.2	268.4	654.1	183.5	120.5	189.6	444.0
Argentina	AMRO	578.6	1133.2	182.7	532.6	513.1	272.6	128.3	258.9	383.1

WHO region: AFRO=Africa; AMRO=Americas; EMRO=Eastern Mediterranean; EURO=Europe; SEARO=South-East Asia; WPRO=Western Pacific.

Other abbreviations: COPD, chronic obstructive pulmonary disease; CVD, cardiovascular disease.

[a] Data were available for 69 individual countries, plus Hong Kong; data for an additional 6 countries were aggregated as Central America, and data for a further 12 countries were aggregated as West Africa. The sample thus consisted of 72 markets, covering 87 countries (69 individual countries and 2 regions of 18 countries) plus the special administrative region of Hong Kong.

© 2025 Wouters OJ et al. JAMA Health Forum.

Table A8. Days of minimum wage required to pay for a course of treatment, by country.

Country	Amoxicillin	Escitalopram	Ibuprofen	Insulin injection (soluble)	Losartan	Paclitaxel	Salbutamol	Tenofovir disoproxil
Argentina	0.3	1.7	0.1	0.9	1.0	66.4	0.2	12.1
Australia	< 0.1	< 0.1	< 0.1	< 0.1	0.6	0.4	< 0.1	2.1
Bangladesh	0.5	1.2	0.1	0.6	0.9	62.3	0.2	9.6
Belarus	0.6	1.9	0.5	0.4	0.4	5.9	0.1	1.0
Belgium	< 0.1	0.1	< 0.1	0.1	0.1	8.8	< 0.1	3.3
Bosnia	0.2	0.5	0.2	0.5	0.4	3.7	< 0.1	2.0
Brazil	0.7	1.5	1.2	0.5	0.4	302.1	0.3	.
Bulgaria	0.3	0.5	0.4	0.4	0.2	3.0	< 0.1	7.6
Canada	< 0.1	0.3	< 0.1	0.1	0.3	0.4	< 0.1	2.6
Central America [a]	0.2	2.8	0.1	0.4	0.3	13.7	0.1	.
Chile	0.1	0.7	< 0.1	0.7	0.1	7.2	< 0.1	13.2
China	0.3	2.2	0.9	0.6	0.8	15.6	0.1	1.0
Colombia	0.3	2.6	0.2	0.4	0.3	7.7	0.1	3.9
Croatia	< 0.1	0.2	0.2	0.2	0.1	25.8	0.1	9.0
Czech Republic	0.1	0.2	< 0.1	0.2	< 0.1	4.6	< 0.1	10.3
Dominican Republic	0.6	4.2	0.6	0.5	1.3	3.7	0.2	2.8
Ecuador	0.3	2.6	0.1	0.4	0.4	13.0	0.2	.
Egypt	0.3	1.5	0.2	0.2	0.8	40.9	< 0.1	3.7
Estonia	0.2	0.3	0.2	0.3	0.1	.	< 0.1	6.4
France	< 0.1	0.1	< 0.1	0.1	< 0.1	0.9	< 0.1	2.7
Germany	< 0.1	< 0.1	< 0.1	0.2	< 0.1	4.8	< 0.1	2.9
Greece	< 0.1	0.4	0.2	0.2	0.2	10.5	< 0.1	5.9
Hong Kong	< 0.1	0.6	< 0.1	0.2	< 0.1	9.2	< 0.1	0.3
Hungary	< 0.1	0.3	0.3	0.2	0.1	36.9	< 0.1	7.2
India	0.9	1.8	0.2	2.5	1.3	65.9	0.3	10.0

© 2025 Wouters OJ et al. JAMA Health Forum.

Indonesia	0.2	6.0	< 0.1	1.5	2.4	63.6	0.5	3.5
Ireland	< 0.1	0.1	< 0.1	< 0.1	0.1	9.4	< 0.1	3.9
Japan	.	0.9	.	0.2	0.2	3.6	< 0.1	4.4
Jordan	0.4	1.1	0.1	0.4	0.8	.	< 0.1	.
Kazakhstan	1.0	2.1	0.4	0.3	1.2	17.9	0.2	0.8
Kuwait	0.8	3.5	0.3	0.7	2.6	.	0.2	.
Latvia	0.2	0.2	0.1	0.3	0.1	4.9	< 0.1	6.6
Lebanon	< 0.1	0.3	< 0.1	< 0.1	0.3	8.8	< 0.1	1.0
Lithuania	0.2	0.1	0.2	0.2	0.1	2.0	< 0.1	6.5
Luxembourg	< 0.1	0.2	< 0.1	< 0.1	< 0.1	.	< 0.1	2.6
Malaysia	0.1	2.4	0.1	0.2	0.3	7.6	0.1	0.8
Mexico	0.9	3.6	0.5	0.6	0.3	16.0	0.2	12.1
Morocco	0.6	1.5	0.2	0.4	0.8	28.6	0.1	3.1
Netherlands	< 0.1	< 0.1	< 0.1	0.1	< 0.1	.	< 0.1	0.6
New Zealand	< 0.1	< 0.1	< 0.1	< 0.1	< 0.1	0.4	< 0.1	0.4
Pakistan	0.3	0.9	< 0.1	0.3	0.6	36.2	0.1	4.4
Peru	0.1	2.2	< 0.1	0.6	0.1	.	0.1	.
Philippines	0.5	6.2	0.3	1.1	1.4	82.2	0.4	5.2
Poland	0.1	0.2	0.1	0.2	0.1	1.1	< 0.1	1.6
Portugal	0.2	0.2	0.2	0.2	0.2	0.9	< 0.1	0.5
Romania	0.1	0.3	0.3	0.3	0.1	5.6	< 0.1	5.8
Russia	0.5	3.9	0.3	0.4	0.4	15.8	0.1	0.6
Saudi Arabia	0.2	0.5	< 0.1	0.1	0.4	5.9	< 0.1	9.5
Serbia	0.1	0.1	0.1	0.3	0.1	2.7	< 0.1	9.3
Slovakia	0.1	0.1	0.1	0.2	< 0.1	3.0	< 0.1	3.8
Slovenia	0.1	0.1	0.2	0.1	< 0.1	16.9	< 0.1	3.9
South Africa	0.1	0.9	0.1	0.2	0.4	10.8	< 0.1	0.6
South Korea	< 0.1	0.2	< 0.1	< 0.1	0.2	7.6	< 0.1	1.2
Spain	< 0.1	0.2	< 0.1	0.2	0.1	16.0	< 0.1	2.0
Sri Lanka	0.5	2.3	0.2	1.6	0.7	80.2	0.3	.

© 2025 Wouters OJ et al. JAMA Health Forum.

Switzerland	< 0.1	0.2	< 0.1	< 0.1	0.1	2.9	< 0.1	4.2
Taiwan	< 0.1	0.3	< 0.1	0.1	0.1	8.6	< 0.1	5.1
Thailand	0.2	3.3	< 0.1	0.5	0.2	82.3	0.2	1.7
Tunisia	0.3	2.7	0.4	0.2	1.9	8.0	0.1	5.8
Türkiye	0.1	0.1	0.1	0.1	0.1	2.0	< 0.1	1.9
UK	< 0.1	< 0.1	< 0.1	< 0.1	< 0.1	25.9	< 0.1	1.1
Uruguay	0.6	0.2	0.2	0.3	0.1	3.8	< 0.1	1.7
USA	< 0.1	< 0.1	< 0.1	0.8	< 0.1	1.2	0.2	1.3
Vietnam	0.2	1.4	0.1	0.2	0.5	19.6	0.2	1.4
West Africa [a]	0.7	3.8	0.4	0.7	3.1	129.5	0.5	3.6

Abbreviations: UK, United Kingdom; USA, United States of America.

[a] Data for 6 countries were aggregated Central America, and data for 12 countries were aggregated as West Africa.

© 2025 Wouters OJ et al. JAMA Health Forum.

Table A9. Main price index results (generic vs. brand-name medicines), by country.

Country	WHO region	Full sample	Brand-name drugs [a]	Generic drugs
Lebanon	EMRO	18.1	10.0	32.6
Australia	WPRO	52.9	47.6	74.4
New Zealand	WPRO	65.4	65.0	58.2
Bangladesh	SEARO	77.7	112.9	124.7
Turkey	EURO	80.7	56.1	137.1
Sweden	EURO	84.5	67.0	75.0
Japan	WPRO	89.5	75.2	117.7
France	EURO	96.1	80.5	238.7
Netherlands	EURO	96.8	61.6	133.7
Pakistan	EMRO	97.6	74.7	167.9
Germany	EURO	100.0	100.0	100.0
South Korea	WPRO	103.4	50.3	229.8
Canada	AMRO	104.3	107.0	146.1
Finland	EURO	112.5	88.8	200.2
Czech Republic	EURO	120.5	122.6	130.9
Estonia	EURO	120.7	60.3	247.6
Lithuania	EURO	121.1	80.7	165.0
Portugal	EURO	124.1	79.3	172.6
UK	EURO	126.5	95.5	149.7
Singapore	WPRO	126.9	80.1	234.6
Norway	EURO	128.8	81.8	195.0
Serbia	EURO	129.9	108.7	303.6
Slovakia	EURO	130.4	105.5	155.7
Hong Kong	WPRO	132.1	83.4	224.7
Poland	EURO	133.1	101.2	183.3
Russia	EURO	133.5	109.0	213.1
China	WPRO	133.6	79.0	307.4
Latvia	EURO	138.7	91.2	235.7
India	SEARO	139.2	155.7	187.9
Luxembourg	EURO	139.5	93.5	168.4
Hungary	EURO	141.0	108.8	151.0
Greece	EURO	143.7	88.2	266.0
Bulgaria	EURO	144.3	122.4	178.0
South Africa	AFRO	150.8	96.7	234.8
Uruguay	AMRO	154.2	118.1	183.9
Ireland	EURO	156.2	115.9	182.0
Belarus	EURO	157.0	134.0	199.0
Spain	EURO	158.8	126.0	185.5
Romania	EURO	162.2	142.8	189.6
Switzerland	EURO	162.7	107.8	269.9

© 2025 Wouters OJ et al. JAMA Health Forum.

Sri Lanka	SEARO	165.3	93.8	202.9
Belgium	EURO	170.8	128.1	188.0
Vietnam	WPRO	172.2	163.3	211.9
Kazakhstan	EURO	174.5	188.5	248.2
Croatia	EURO	177.2	135.9	274.9
Taiwan	WPRO	180.3	115.5	348.9
Tunisia	EMRO	188.3	178.2	260.6
Slovenia	EURO	189.6	92.8	215.7
Bosnia	EURO	191.1	137.0	295.5
Saudi Arabia	EMRO	195.1	119.9	386.0
Malaysia	WPRO	195.4	141.4	244.7
Kuwait	EMRO	201.1	118.0	314.3
Peru	AMRO	208.0	160.8	315.5
Egypt	EMRO	208.4	167.4	321.8
Austria	EURO	222.5	163.5	306.4
Jordan	EMRO	224.9	176.6	354.4
Morocco	EMRO	238.4	186.3	381.6
Italy	EURO	242.4	377.1	229.4
UAE	EMRO	262.1	137.2	485.7
Chile	AMRO	271.4	156.2	518.7
USA	AMRO	298.2	542.3	170.6
West Africa	AFRO	304.9	278.7	406.5
Ecuador	AMRO	309.3	187.2	511.8
Brazil	AMRO	313.5	195.9	512.0
Colombia	AMRO	316.0	186.8	541.8
Philippines	WPRO	321.5	166.5	575.5
Central America	AMRO	385.4	206.7	634.6
Indonesia	SEARO	393.2	230.7	787.3
Thailand	SEARO	399.8	290.7	999.5
Dominican Republic	AMRO	412.1	274.6	707.9
Mexico	AMRO	432.1	235.1	738.3
Argentina	AMRO	578.6	437.8	950.4

WHO region: AFRO=Africa; AMRO=Americas; EMRO=Eastern Mediterranean; EURO=Europe; SEARO=South-East Asia; WPRO=Western Pacific.

Other abbreviations: UAE, United Arab Emirates; UK, United Kingdom; USA, United States of America.

[a] Includes off-patent originators.

© 2025 Wouters OJ et al. JAMA Health Forum.

Table A10. Price index by drug category (median index in each WHO region).

WHO Region	Full sample	Antibiotics	Asthma and COPD	Cancer	CVD	Diabetes	Hepatitis B and C	Migraine and pain	Mental Health
AFRO	227.9	214.3	121.6	118.6	285.5	102.6	14.4	109.1	195.9
AMRO	311.4	371.9	140.9	181.6	379.0	179.5	95.8	208.1	387.2
EMRO	201.1	257.2	100.5	182.2	263.3	95.4	19.0	82.5	222.5
EURO	138.7	181.2	86.4	118.2	136.5	90.5	96.6	106.4	116.4
SEARO	165.3	242.8	49.5	90.4	244.6	87.6	33.1	54.6	131.8
WPRO	132.1	148.7	74.2	84.8	140.3	68.4	30.0	76.0	188.8

WHO region: AFRO=Africa; AMRO=Americas; EMRO=Eastern Mediterranean; EURO=Europe; SEARO=South-East Asia; WPRO=Western Pacific.

Other abbreviations: COPD, chronic obstructive pulmonary disease; CVD, cardiovascular disease.

© 2025 Wouters OJ et al. JAMA Health Forum.

Table A11. Share of brand-name, generic, and over-the-counter drugs in each market by value and volume, 2022 [a]

Country	Value of products				Volume of products			
	Brand-name drugs	Generic drugs	Over-the-counter drugs	Unknown	Brand-name drugs	Generic drugs	Over-the-counter drugs	Unknown
Total	67%	30%	2%	<1%	14%	70%	13%	2%
Argentina	23%	72%	4%	<1%	10%	77%	13%	<1%
Australia	65%	29%	5%	<1%	19%	65%	15%	1%
Austria	69%	29%	2%	<1%	26%	65%	8%	1%
Bangladesh	3%	96%	0%	1%	2%	97%	0%	1%
Belarus	19%	67%	14%	<1%	3%	51%	45%	1%
Belgium	76%	23%	0%	<1%	32%	68%	0%	<1%
Bosnia	42%	49%	9%	<1%	18%	63%	18%	1%
Brazil	19%	70%	11%	<1%	9%	70%	20%	1%
Bulgaria	62%	30%	8%	<1%	18%	53%	22%	7%
Canada	66%	29%	5%	<1%	13%	62%	25%	<1%
Central America [b]	34%	58%	8%	<1%	9%	68%	22%	1%
Chile	29%	61%	10%	<1%	6%	69%	25%	<1%
China	40%	59%	1%	1%	11%	68%	4%	16%
Colombia	12%	60%	28%	<1%	3%	49%	49%	<1%
Croatia	63%	32%	5%	<1%	36%	52%	11%	1%
Czech Republic	66%	29%	4%	<1%	23%	65%	12%	<1%
Dominican Republic	18%	78%	4%	<1%	5%	78%	17%	1%
Ecuador	24%	71%	5%	<1%	9%	76%	12%	3%
Egypt	44%	54%	0%	1%	27%	71%	0%	2%
Estonia	56%	32%	11%	<1%	24%	54%	21%	<1%
Finland	68%	27%	4%	<1%	23%	64%	14%	<1%
France	62%	37%	<1%	<1%	16%	83%	<1%	1%
Germany	67%	29%	4%	<1%	11%	68%	21%	<1%

© 2025 Wouters OJ et al. JAMA Health Forum.

Greece	61%	33%	5%	1%	36%	50%	12%	1%
Hong Kong	73%	19%	8%	<1%	13%	47%	40%	<1%
Hungary	64%	35%	<1%	<1%	25%	74%	1%	1%
India	15%	80%	4%	<1%	7%	78%	14%	<1%
Indonesia	27%	70%	3%	<1%	4%	82%	11%	2%
Ireland	68%	25%	6%	<1%	24%	55%	20%	1%
Italy	73%	27%	0%	<1%	34%	64%	0%	2%
Japan	72%	26%	1%	<1%	25%	71%	3%	1%
Jordan	40%	60%	0%	<1%	24%	75%	0%	1%
Kazakhstan	31%	56%	13%	<1%	5%	61%	32%	1%
Kuwait	58%	41%	0%	<1%	36%	63%	0%	<1%
Latvia	52%	34%	13%	<1%	18%	55%	23%	4%
Lebanon	36%	63%	0%	1%	23%	75%	0%	2%
Lithuania	62%	27%	11%	<1%	24%	52%	23%	1%
Luxembourg	79%	18%	3%	<1%	47%	43%	11%	<1%
Malaysia	53%	25%	21%	<1%	6%	36%	58%	<1%
Mexico	38%	53%	9%	<1%	11%	64%	25%	1%
Morocco	36%	64%	0%	<1%	31%	69%	0%	<1%
Netherlands	49%	50%	1%	<1%	13%	85%	1%	<1%
New Zealand	69%	28%	4%	<1%	17%	74%	8%	<1%
Norway	65%	33%	2%	<1%	20%	63%	16%	1%
Pakistan	23%	76%	0%	<1%	34%	65%	0%	1%
Peru	22%	64%	14%	<1%	6%	64%	30%	1%
Philippines	36%	56%	8%	<1%	10%	60%	29%	1%
Poland	54%	35%	11%	<1%	19%	58%	23%	<1%
Portugal	61%	39%	1%	<1%	28%	70%	1%	1%
Romania	63%	26%	10%	<1%	30%	47%	22%	1%
Russia	47%	38%	15%	<1%	8%	45%	46%	<1%
Saudi Arabia	49%	51%	0%	<1%	21%	79%	0%	1%
Serbia	49%	45%	6%	<1%	19%	71%	7%	3%

© 2025 Wouters OJ et al. JAMA Health Forum.

Singapore	77%	14%	9%	<1%	22%	33%	45%	<1%
Slovakia	62%	34%	4%	1%	20%	68%	10%	2%
Slovenia	68%	30%	2%	<1%	32%	54%	13%	<1%
South Africa	34%	57%	9%	1%	6%	74%	18%	2%
South Korea	54%	42%	4%	<1%	24%	49%	27%	1%
Spain	68%	32%	<1%	<1%	24%	75%	<1%	1%
Sri Lanka	16%	84%	0%	<1%	8%	90%	0%	2%
Sweden	67%	29%	4%	<1%	14%	72%	14%	1%
Switzerland	64%	32%	4%	<1%	21%	63%	15%	1%
Taiwan	76%	22%	1%	<1%	29%	55%	14%	1%
Thailand	46%	50%	4%	<1%	6%	69%	25%	<1%
Tunisia	35%	61%	4%	<1%	11%	76%	13%	<1%
Türkiye	51%	49%	<1%	1%	26%	72%	<1%	3%
UAE	58%	42%	0%	<1%	29%	70%	0%	1%
UK	63%	35%	1%	<1%	16%	75%	8%	1%
Uruguay	22%	73%	5%	1%	8%	80%	12%	1%
USA	82%	17%	1%	<1%	6%	77%	16%	<1%
Vietnam	40%	54%	5%	<1%	7%	75%	17%	1%
West Africa [b]	21%	78%	0%	2%	12%	86%	0%	2%

Abbreviations: UAE, United Arab Emirates; UK, United Kingdom; USA, United States of America.

[a] Percentages may not add up to 100% due to rounding.
[b] Data for 6 countries were aggregated as Central America, and data for 12 countries were aggregated as West Africa.

© 2025 Wouters OJ et al. JAMA Health Forum.

Table A12. Days of minimum wage required to pay for a course of treatment, by WHO region.

WHO Region		Amoxicillin	Escitalopram	Ibuprofen	Insulin Injection (Soluble)	Losartan	Paclitaxel	Salbutamol	Tenofovir Disoproxil
	Median	0.44	2.36	0.24	0.45	1.74	70.19	0.28	2.09
AFRO	[Q1, Q3]	.	.	.	.	.	.	.	.
	[Min, Max]	[0.14, 0.75]	[0.94, 3.79]	[0.11, 0.37]	[0.24, 0.66]	[0.39, 3.09]	[10.84, 129.54]	[0.07, 0.49]	[0.59, 3.58]
	Median	0.28	1.92	0.14	0.49	0.33	7.67	0.16	3.37
AMRO	[Q1, Q3]	[0.12, 0.57]	[0.58, 2.67]	[0.07, 0.27]	[0.41, 0.65]	[0.12, 0.40]	[3.79, 14.86]	[0.10, 0.20]	[2.40, 12.09]
	[Min, Max]	[0.04, 0.90]	[0.07, 4.20]	[0.02, 1.17]	[0.12, 0.94]	[0.07, 1.26]	[0.44, 302.13]	[0.03, 0.33]	[1.32, 13.22]
	Median	0.29	1.28	0.16	0.28	0.80	18.69	0.09	4.06
EMRO	[Q1, Q3]	[0.25, 0.43]	[0.82, 1.79]	[0.07, 0.26]	[0.17, 0.38]	[0.55, 1.10]	[8.22, 34.27]	[0.07, 0.13]	[3.22, 5.47]
	[Min, Max]	[0.05, 0.80]	[0.30, 3.46]	[0.04, 0.38]	[0.03, 0.66]	[0.32, 2.58]	[5.85, 40.93]	[0.01, 0.16]	[0.95, 9.50]
	Median	0.11	0.21	0.14	0.19	0.12	4.86	0.03	3.54
EURO	[Q1, Q3]	[0.08, 0.18]	[0.12, 0.28]	[0.08, 0.18]	[0.11, 0.27]	[0.09, 0.15]	[2.93, 15.83]	[0.03, 0.05]	[1.86, 6.41]
	[Min, Max]	[0.03, 1.00]	[0.05, 3.88]	[0.02, 0.46]	[0.07, 0.52]	[0.03, 1.23]	[0.89, 36.85]	[0.01, 0.16]	[0.50, 10.34]
	Median	0.46	2.25	0.11	1.48	0.94	65.88	0.29	6.54
SEARO	[Q1, Q3]	[0.24, 0.54]	[1.80, 3.27]	[0.09, 0.17]	[0.58, 1.56]	[0.66, 1.31]	[63.62, 80.22]	[0.24, 0.30]	[3.03, 9.71]
	[Min, Max]	[0.16, 0.87]	[1.24, 6.05]	[0.06, 0.21]	[0.47, 2.53]	[0.23, 2.37]	[62.31, 82.27]	[0.18, 0.48]	[1.71, 10.04]
	Median	0.06	0.76	0.08	0.16	0.26	8.12	0.04	1.28
WPRO	[Q1, Q3]	[0.03, 0.20]	[0.25, 1.99]	[0.03, 0.12]	[0.09, 0.21]	[0.15, 0.53]	[4.56, 13.98]	[0.03, 0.10]	[0.83, 3.82]
	[Min, Max]	[0.02, 0.47]	[0.01, 6.18]	[0.01, 0.90]	[0.06, 1.07]	[0.01, 1.36]	[0.38, 82.16]	[0.01, 0.40]	[0.31, 5.21]

* No interquartile range reported for AFRO.

WHO region: AFRO=Africa; AMRO=Americas; EMRO=Eastern Mediterranean; EURO=Europe; SEARO=South-East Asia; WPRO=Western Pacific.

© 2025 Wouters OJ et al. JAMA Health Forum.

eAppendix 6. Main results without purchasing power parity adjustments

Table A13. Price index results broken down by disease area.

Country	WHO Region	Full sample (N=549)	Antibiotics (N=58)	Asthma and COPD (N=20)	Cancer (N=68)	CVD (N=143)	Diabetes (N=17)	Hepatitis B and C (N=13)	Migraine and pain (N=11)	Mental health (N=23)
Pakistan	EMRO	30.3	46.7	13.6	49.4	37.2	12.5	5.2	8.0	25.0
Türkiye	EURO	30.5	30.2	16.2	23.5	35.3	15.1	17.9	15.7	32.6
Bangladesh	SEARO	31.6	54.9	14.5	18.2	37.5	24.5	11.5	9.0	24.1
India	SEARO	50.0	52.6	17.8	32.3	62.8	31.6	6.5	12.8	47.4
Sri Lanka	SEARO	53.8	140.1	15.6	28.5	79.5	21.9	.	35.7	39.7
Belarus	EURO	57.0	42.3	44.5	32.2	66.5	55.0	6.4	32.0	74.7
Egypt	EMRO	59.3	73.7	30.2	56.9	65.5	25.4	6.6	18.0	49.8
Serbia	EURO	67.5	55.0	52.3	63.5	65.4	41.1	33.0	49.4	47.8
Australia	WPRO	68.8	75.6	63.2	47.7	86.8	50.0	71.1	63.9	87.4
Vietnam	WPRO	69.4	60.2	41.6	47.4	91.2	44.8	3.9	24.9	80.3
Russia	EURO	69.5	100.1	46.1	55.3	85.0	56.8	13.3	42.6	144.1
Tunisia	EMRO	70.7	74.7	36.4	67.8	98.9	44.3	1.4	31.0	83.6
Poland	EURO	73.1	69.5	48.3	58.8	95.0	55.4	48.5	66.7	71.8
Kazakhstan	EURO	74.9	85.0	67.8	65.6	77.3	48.7	6.7	32.6	113.8
Bulgaria	EURO	77.7	85.2	55.1	63.4	93.1	61.7	66.1	73.9	69.8
Romania	EURO	81.0	107.6	43.2	85.9	87.1	62.5	73.1	92.8	70.9
Lithuania	EURO	81.5	100.2	47.7	65.4	74.6	55.4	20.6	98.7	83.6
Hungary	EURO	81.7	79.2	54.3	70.9	68.7	54.6	57.0	92.4	51.2
New Zealand	WPRO	82.5	101.8	44.1	70.3	81.3	59.7	71.0	26.9	42.3
Czech Republic	EURO	86.9	119.6	50.7	94.7	74.8	73.2	79.6	71.0	83.1
Japan	WPRO	88.7	93.5	73.8	59.9	103.8	67.9	66.8	98.1	187.4
South Korea	WPRO	88.9	91.2	48.5	44.7	121.0	40.8	23.9	53.4	78.6

© 2025 Wouters OJ et al. JAMA Health Forum.

South Africa	AFRO	91.2	72.6	51.1	60.3	128.5	36.2	12.5	46.9	74.5
Malaysia	WPRO	91.6	151.4	36.5	62.5	126.7	42.9	17.0	41.7	206.9
Slovakia	EURO	92.5	122.1	51.4	78.4	81.7	68.0	72.9	74.6	49.8
France	EURO	93.3	181.9	62.2	80.4	109.3	62.3	80.4	87.3	89.0
Portugal	EURO	93.4	68.7	59.0	58.3	106.6	65.2	16.9	91.7	97.0
Bosnia	EURO	94.2	68.6	73.1	60.3	85.5	66.5	28.1	62.1	50.1
Sweden	EURO	95.7	181.0	67.9	69.1	83.1	61.9	71.8	54.9	63.5
Estonia	EURO	96.4	343.6	63.1	48.5	85.4	59.2	43.4	98.0	72.6
Latvia	EURO	98.5	155.2	64.2	72.1	79.6	64.9	12.5	92.8	76.3
Germany	EURO	100.0	100.0	100.0	100.0	100.0	100.0	100.0	100.0	100.0
Netherlands	EURO	101.3	269.6	53.0	125.9	86.8	54.5	50.9	88.9	105.1
China	WPRO	103.1	424.5	100.6	65.3	111.7	54.8	4.1	115.4	217.9
Greece	EURO	105.3	133.3	81.2	71.0	141.8	68.4	98.7	102.3	115.8
Croatia	EURO	106.6	162.3	61.5	127.5	104.5	65.1	98.0	76.0	74.8
Singapore	WPRO	108.1	220.2	85.6	82.0	110.7	42.3	15.0	64.8	261.0
Taiwan	WPRO	119.2	90.2	46.9	74.7	115.0	79.2	29.8	59.8	86.0
Morocco	EMRO	122.4	143.8	51.6	132.2	153.5	67.2	9.7	77.0	128.2
Finland	EURO	124.5	381.2	84.7	119.2	104.0	73.1	133.8	117.8	110.1
Peru	AMRO	125.9	93.2	59.1	91.9	161.2	98.8	17.1	86.4	237.0
Canada	AMRO	127.6	293.6	125.3	114.1	129.5	105.6	117.2	73.3	131.1
Hong Kong	WPRO	128.6	165.6	58.3	77.8	124.5	58.4	21.1	36.0	109.3
Uruguay	AMRO	132.3	101.5	112.4	90.8	102.1	78.9	15.1	115.7	82.2
Spain	EURO	132.4	147.8	68.0	135.6	134.2	75.9	124.4	69.4	106.3
Lebanon	EMRO	133.5	381.0	69.0	111.4	192.9	93.0	42.6	68.6	164.2
Jordan	EMRO	134.4	239.4	56.3	116.9	169.3	70.8	.	69.6	112.0
Colombia	AMRO	137.8	111.7	65.4	73.4	158.4	74.5	52.5	96.2	220.0
UK	EURO	139.0	287.1	75.0	139.3	134.1	75.7	140.9	51.9	105.1
West Africa	AFRO	143.6	145.7	74.8	64.4	168.9	68.7	3.6	66.3	126.6
Slovenia	EURO	144.1	240.3	63.3	104.4	83.1	51.8	69.2	80.0	66.0

© 2025 Wouters OJ et al. JAMA Health Forum.

Saudi Arabia	EMRO	149.6	188.7	83.7	107.6	173.0	73.5	102.2	65.3	288.7
Philippines	WPRO	154.2	222.0	74.7	100.8	169.8	58.9	14.2	78.7	271.0
Norway	EURO	155.1	314.8	87.4	136.4	107.8	74.1	131.4	90.5	99.9
Luxembourg	EURO	162.8	179.3	100.9	116.8	195.9	87.0	111.9	135.6	136.0
Ireland	EURO	165.8	239.9	91.7	142.3	145.0	87.7	101.3	120.4	138.2
Thailand	SEARO	170.3	123.8	74.6	122.8	234.0	93.3	16.1	23.2	110.6
Belgium	EURO	170.8	156.4	89.3	160.4	159.9	89.4	113.6	95.6	127.0
Indonesia	SEARO	177.8	110.4	143.6	93.4	172.4	68.4	22.8	123.5	107.9
Ecuador	AMRO	181.1	173.0	57.3	122.7	242.8	103.1	.	115.3	330.9
Chile	AMRO	181.4	434.5	79.7	112.3	263.7	132.9	82.5	150.3	255.9
Brazil	AMRO	200.1	357.8	64.5	190.7	184.0	71.3	75.3	205.2	202.3
Italy	EURO	207.7	234.7	90.3	191.5	169.4	108.0	166.4	155.4	162.9
Kuwait	EMRO	209.8	332.3	107.7	104.5	303.9	93.4	.	70.3	275.6
Austria	EURO	223.1	679.9	137.9	237.1	212.1	128.6	162.8	122.1	221.0
Switzerland	EURO	228.6	659.1	169.6	191.1	261.1	119.9	185.5	190.5	282.6
Dominican Republic	AMRO	234.6	358.6	125.6	101.7	289.7	110.8	2.0	170.2	300.8
UAE	EMRO	243.7	273.9	132.2	168.9	297.1	110.5	151.1	109.5	375.6
Central America	AMRO	268.9	314.4	145.8	171.1	377.6	135.2	6.7	174.0	403.2
Mexico	AMRO	284.5	310.2	129.3	176.1	430.6	121.3	79.4	125.0	292.5
Argentina	AMRO	363.1	692.3	116.9	317.9	329.0	174.7	88.2	166.7	245.6
USA	AMRO	407.5	151.8	639.3	250.7	468.8	647.6	89.9	58.9	139.7

© 2025 Wouters OJ et al. JAMA Health Forum.

Table A14. Main price index results (generic vs. brand-name medicines), by country.

Country	WHO region	Full sample	Brand-name medicines [a]	Generic medicines
Pakistan	EMRO	30.3	26.1	52.1
Türkiye	EURO	30.5	22.3	51.8
Bangladesh	SEARO	31.6	46.5	50.6
India	SEARO	50.0	56.0	67.6
Sri Lanka	SEARO	53.8	30.4	66.6
Belarus	EURO	57.0	48.1	71.9
Egypt	EMRO	59.3	47.8	90.8
Serbia	EURO	67.5	56.5	158.1
Australia	WPRO	68.8	62.2	96.9
Vietnam	WPRO	69.4	66.1	85.5
Russia	EURO	69.5	56.7	112.3
Tunisia	EMRO	70.7	115.0	97.7
Poland	EURO	73.1	55.5	100.8
Kazakhstan	EURO	74.9	80.9	106.5
Bulgaria	EURO	77.7	65.8	96.0
Romania	EURO	81.0	71.9	94.7
Lithuania	EURO	81.5	54.2	111.2
Hungary	EURO	81.7	63.7	87.5
New Zealand	WPRO	82.5	82.1	73.2
Czech Republic	EURO	86.9	88.3	94.5
Japan	WPRO	88.7	74.6	116.3
South Korea	WPRO	88.9	43.2	197.5
South Africa	AFRO	91.2	61.7	142.0
Malaysia	WPRO	91.6	67.0	114.9
Slovakia	EURO	92.5	74.7	110.7
France	EURO	93.3	78.1	230.9
Portugal	EURO	93.4	59.8	129.9
Bosnia	EURO	94.2	67.4	145.7
Sweden	EURO	95.7	76.1	85.1
Estonia	EURO	96.4	48.4	198.2
Latvia	EURO	98.5	65.2	166.7
Germany	EURO	100.0	100.0	100.0
Netherlands	EURO	101.3	65.4	139.5
China	WPRO	103.1	60.9	237.2
Greece	EURO	105.3	65.4	194.5
Croatia	EURO	106.6	81.8	165.6
Singapore	WPRO	108.1	68.6	200.2
Taiwan	WPRO	119.2	84.5	230.6
Morocco	EMRO	122.4	95.6	196.1

© 2025 Wouters OJ et al. JAMA Health Forum.

Finland	EURO	124.5	98.4	221.5
Peru	AMRO	125.9	97.6	191.0
Canada	AMRO	127.6	131.1	178.9
Hong Kong	WPRO	128.6	93.4	218.8
Uruguay	AMRO	132.3	97.3	157.9
Spain	EURO	132.4	105.1	154.7
Lebanon	EMRO	133.5	74.9	240.2
Jordan	EMRO	134.4	105.7	212.0
Colombia	AMRO	137.8	85.8	236.8
UK	EURO	139.0	105.0	164.0
West Africa	AFRO	143.6	130.3	191.5
Slovenia	EURO	144.1	70.7	164.0
Saudi Arabia	EMRO	149.6	90.9	296.2
Philippines	WPRO	154.2	88.8	275.8
Norway	EURO	155.1	98.4	233.5
Luxembourg	EURO	162.8	109.5	196.9
Ireland	EURO	165.8	122.9	193.3
Thailand	SEARO	170.3	122.1	426.1
Belgium	EURO	170.8	128.0	187.9
Indonesia	SEARO	177.8	104.1	356.5
Ecuador	AMRO	181.1	109.5	299.9
Chile	AMRO	181.4	104.5	345.2
Brazil	AMRO	200.1	125.3	327.4
Italy	EURO	207.7	324.2	196.6
Kuwait	EMRO	209.8	126.1	327.9
Austria	EURO	223.1	163.9	307.1
Switzerland	EURO	228.6	151.5	379.5
Dominican Republic	AMRO	234.6	155.5	403.5
UAE	EMRO	243.7	127.6	451.9
Central America	AMRO	268.9	144.5	443.1
Mexico	AMRO	284.5	154.6	486.8
Argentina	AMRO	363.1	269.3	606.5
USA	AMRO	407.5	741.6	233.3

© 2025 Wouters OJ et al. JAMA Health Forum.

Table A15. Price index by drug category (median index in each WHO region).

WHO Region	Full sample	Antibiotics	Asthma and COPD	Cancer	CVD	Diabetes	Hepatitis B and C	Migraine and pain	Mental Health
AFRO	117.4	109.1	63.0	62.4	148.7	52.5	8.1	56.6	100.6
AMRO	190.8	301.9	114.7	118.4	253.3	108.2	75.3	120.4	241.3
EMRO	133.5	188.7	56.3	107.6	169.3	70.8	9.7	68.6	128.2
EURO	96.4	147.8	63.3	80.4	93.1	65.1	72.9	88.9	89.0
SEARO	53.8	110.4	17.8	32.3	79.5	31.6	13.8	23.2	47.4
WPRO	91.6	101.8	58.3	65.3	111.7	54.8	21.1	59.8	109.3

WHO region: AFRO=Africa; AMRO=Americas; EMRO=Eastern Mediterranean; EURO=Europe; SEARO=South-East Asia; WPRO=Western Pacific.

Other abbreviations: COPD, chronic obstructive pulmonary disease; CVD, cardiovascular disease.

© 2025 Wouters OJ et al. JAMA Health Forum.
