# Supplementary material for: Prices and Affordability of Essential Medicines in 72 Low-, Middle-, and High-Income Markets
Source: JAMA Health Forum. 2025 Aug 15;6(8):e252043. doi: 10.1001/jamahealthforum.2025.2043 (PMC12357183; doi:10.1001/jamahealthforum.2025.2043)
Supplement: Supplement 2. — Data Sharing Statement [file jamahealthforum-e252043-s002.pdf]

Data Sharing Statement

Wouters. Prices and Affordability of Essential Medicines in 72 Low-, Middle-, and High-Income Markets. *JAMA Health Forum*. Published August 15, 2025.
doi:10.1001/jamahealthforum.2025.2043

Data
Data available: No

Additional Information
Explanation for why data not available: Data on drug prices and volumes were sourced from IQVIA. All other data were obtained from publicly available sources.
